# Optimizing infectious disease mitigation under dynamic conditions

**DOI:** 10.1073/pnas.2527395123

**Published:** 2026-08-03

**Authors:** Laura Müller, Fabio Sartori, Jonas Dehning, Maximilian F. Eggl, Viola Priesemann

**Affiliations:** ^a^ Max-Planck-Institute for Dynamics and Self-Organization, Göttingen 37077, Germany; ^b^ Institute for the Dynamics of Complex Systems, University of Göttingen, Göttingen 37077, Germany; ^c^ Chair of Sociology and Computational Social Science, Karlsruhe Institute, Karlsruhe Institute of Technology, Karlsruhe 76131, Germany; ^d^ Institute of Neuroscience, Consejo Superior de Investigaciones Científicas - Universidad de Miguel Hernandez, Alicante 03550, Spain; ^e^ Institute of Experimental Epileptology and Cognition Research, University of Bonn Medical Center, Bonn 53127, Germany

**Keywords:** pandemic mitigation, optimal control, seasonality, public health, optimization

## Abstract

Mitigation measures are essential for controlling the spread of infectious diseases during pandemics and epidemics, but they impose considerable societal, individual, and economic costs. We developed a general framework that combines simulation of disease dynamics with optimal control to determine mitigation strategies that balance infection and mitigation costs. Optimizing this trade-off, we identified three surprising effects: first, assuming a constant reproduction number $R_{0}$, the optimal response is typically “all-or-nothing”: depending on disease severity, either strict mitigation or none at all is optimal, with intermediate levels emerging only in restricted regimes that we characterize analytically. Second, under seasonal variations, optimal mitigation is stricter during winter. Interestingly, a single wave of infections still arises in spring, replacing the autumn/winter waves known for classical influenza. Third, during steady vaccination campaigns, even optimal mitigation can result in transient infection waves. Finally, we quantify the cost of delayed mitigation onset and show that even short delays can substantially increase total costs—if the disease is severe. Overall, our framework is easily applicable to general and complex settings and thereby presents a versatile tool to explore optimal mitigation strategies for endemic and pandemic infectious disease.

## Significance

Managing a pandemic requires balancing competing costs: interventions such as mask mandates and lockdowns reduce infections but disrupt economies and social life, while uncontrolled spread imposes treatment costs and loss of productivity and well-being. We present a general framework for identifying optimal strategies that balance these trade-offs under realistic conditions, including seasonality and vaccination. We uncover three key principles: i) interventions are best applied either very strictly or not at all, depending on disease severity; ii) anticipating seasonal changes shifts large outbreaks away from winter, reducing their impact; and iii) even with optimal mitigation, small infection waves can arise during vaccination. By providing both insights and practical tools, our approach offers a foundation for designing responses to future epidemics.

During pandemics like COVID-19, societies face the challenge of implementing measures to mitigate disease spread. These measures, however, come at considerable cost, including economic repercussions (1) as well as social and psychological burdens (2). Still, they can be necessary to reduce the number of new infections, prevent healthcare systems from becoming overwhelmed, and minimize sick leave and preventable deaths. This trade-off raises the fundamental question in pandemic response: what is the optimal mitigation stringency, and how does it change over time and under different conditions? Determining the optimal, time-dependent level of mitigation requires quantifying and balancing infection costs against mitigation costs. This challenge becomes especially complex in long-term pandemic scenarios.

While the optimization of vaccination strategies has been extensively studied (3–6), vaccines are often unavailable during the early phase of a pandemic, if at all. Our study thus centers on mitigation relying on nonpharmaceutical interventions (NPIs) such as social distancing, lockdowns, and mask mandates, including both voluntary and mandatory measures.

A large and diverse body of work has addressed optimal mitigation strategies for infectious diseases from multiple perspectives, including epidemiology, complex systems, and economics. Here, we provide a selective overview of representative methodological approaches rather than an exhaustive survey. Several studies explore mitigation strategies by comparing a limited set of fixed scenarios (7–11). Others use systematic optimization methods based on optimal control theory (12–23) or model predictive control (24–26). Another approach is game theory (27, 28). More recently, machine learning algorithms, such as reinforcement learning, have been explored in addition to the classical approaches (29–32). Each class of methods has its strengths and limitations: classical optimization methods offer interpretability, while machine learning approaches may provide more flexibility and adaptability, though their optimization processes can be less transparent.

For NPIs, a constant level of mitigation measures is optimal during most of the pandemic if one assumes a constant basic reproduction number (14). If, however, the goals, disease parameters, boundary conditions, or cost functions are more complex, studies reach different conclusions. For instance, a strong lockdown followed by gradual relaxation is optimal in many settings influenced by real-world scenarios, whereas premature relaxation can result in large secondary waves (7, 26, 33). If maintained at low numbers, infections can be kept under control with only mild measures complemented by test-trace-and-isolate strategies (7, 34, 35). Additionally, early intervention at the beginning of a pandemic is crucial to prevent large outbreaks and reduce costs (15, 25, 33). Some studies find that for time-limited pandemics where individuals can only get infected once, there is an abrupt transition in the optimal strategy depending on parameters such as altruism and initial infections (27), the cost of social distancing and epidemic duration (36), value of life (19, 22), or population size, infection cost, and optimization time (28).

Despite this progress, important gaps remain. Most existing studies assume a constant or only simply varying basic reproduction number. One study included a linearly increasing reproduction number, for which optimal control measures also need to intensify correspondingly (14). Similarly, mitigation efforts can be relaxed when reproduction numbers decline due to vaccination (14, 21, 37). However, even those scenarios where the reproduction number is varied are still relatively simple. Additionally, most optimizations have been performed using variants of the SIR model without waning immunity, or by assuming that vaccination is distributed sufficiently quickly and is long-lasting. However, for some infectious pathogens, no effective or long-lasting vaccines exist (e.g., HIV, Epstein–Barr virus, SARS-CoV-2, and certain bacterial infections like gonorrhea). For these, long-term optimization of mitigation strategies might become necessary. This requires developing a versatile optimization framework for mitigation in long-term dynamical scenarios.

In this work, we build a comprehensive framework that combines simulation of disease dynamics with optimal control for determining optimal mitigation strategies by minimizing the total cost of a pandemic, consisting of mitigation and infection costs. We introduce general cost functions whose shapes follow basic assumptions about decision-making and that are defined to include all direct and indirect costs associated with mitigation measures and infection, encompassing economic, psychological, and other related impacts. Our framework directly optimizes the total cost numerically with automatic differentiation, rather than relying on classical adjoint-based optimal control formulations such as Pontryagin’s maximum principle. While the problems studied here can in principle also be formulated within classical optimal control frameworks (18–20), such approaches typically require the derivation of adjoint (costate) equations and the solution of coupled forward–backward systems, which can make extensions to more complex models or cost structures less straightforward.

By contrast, recent advances in computational tools—particularly automatic differentiation and high-performance numerical libraries such as JAX (38) and Diffrax (39)—enable a fully numerical implementation that can efficiently handle high-dimensional control problems. This approach falls within the class of direct optimal control methods. It allows for efficient end-to-end, i.e., joint simulation and optimization, while maintaining a high degree of flexibility. We provide a flexible, open-source framework implemented in Python that allows users to easily specify compartmental epidemiological models, modify cost functions, and compute optimal mitigation strategies. It is designed to make it straightforward to incorporate additional epidemiological features and explore a wide range of scenarios. In particular, extending the model (for example by modifying the SIR dynamics or incorporating additional compartments) can be achieved by directly adapting the governing equations in code, without requiring the explicit derivation of problem-specific adjoint equations. This lowers the barrier to entry and facilitates rapid exploration of alternative modeling assumptions, especially for users without specialized expertise in optimal control theory.

This flexibility enables the exploration of a wide variety of scenarios. We first investigate the impact of seasonal fluctuations in the reproduction number on the optimal mitigation strategy and compare it to alternative strategies. Next, we simulate vaccination campaigns to assess how increased population immunity through vaccination can allow for a decrease in mitigation measures. Finally, we analyze the consequences of delayed mitigation efforts at the start of a new pandemic. Our analysis quantifies key outcomes, including the total number of infections, the required intensity of measures, and the overall costs associated with disease management. Across these scenarios, we identify two structural features of the optimal strategy. The first is an “all-or-nothing” dependence on disease severity, which we characterize analytically and whose regime of validity we delimit; we further show that this structure softens into a three-regime pattern when the infection cost has a hospitalization kink. The second is a quarter-period phase shift between mitigation and prevalence under seasonal forcing, which is robust across a wide range of seasonality shapes.

## 1. Materials and Methods

Our approach is to define a cost function (Section 1.1) that depends on the disease dynamics (Section 1.2) and mitigation measures, and optimize the resulting system to minimize the overall integrated costs (Section 1.3). In more detail:

### 1.1. Cost Functions.

The total cost of infectious disease mitigation comprises two components: the cost associated with infections and the cost of mitigation. Both components are formulated to include all direct and indirect costs. Infection-related costs include, for example, medical treatment and loss of productivity, whereas mitigation costs cover not only implementation expenses (e.g., protective equipment) but also broader economic and social effects such as reduced economic activity and mental or social burdens.

**Fig. 1.**
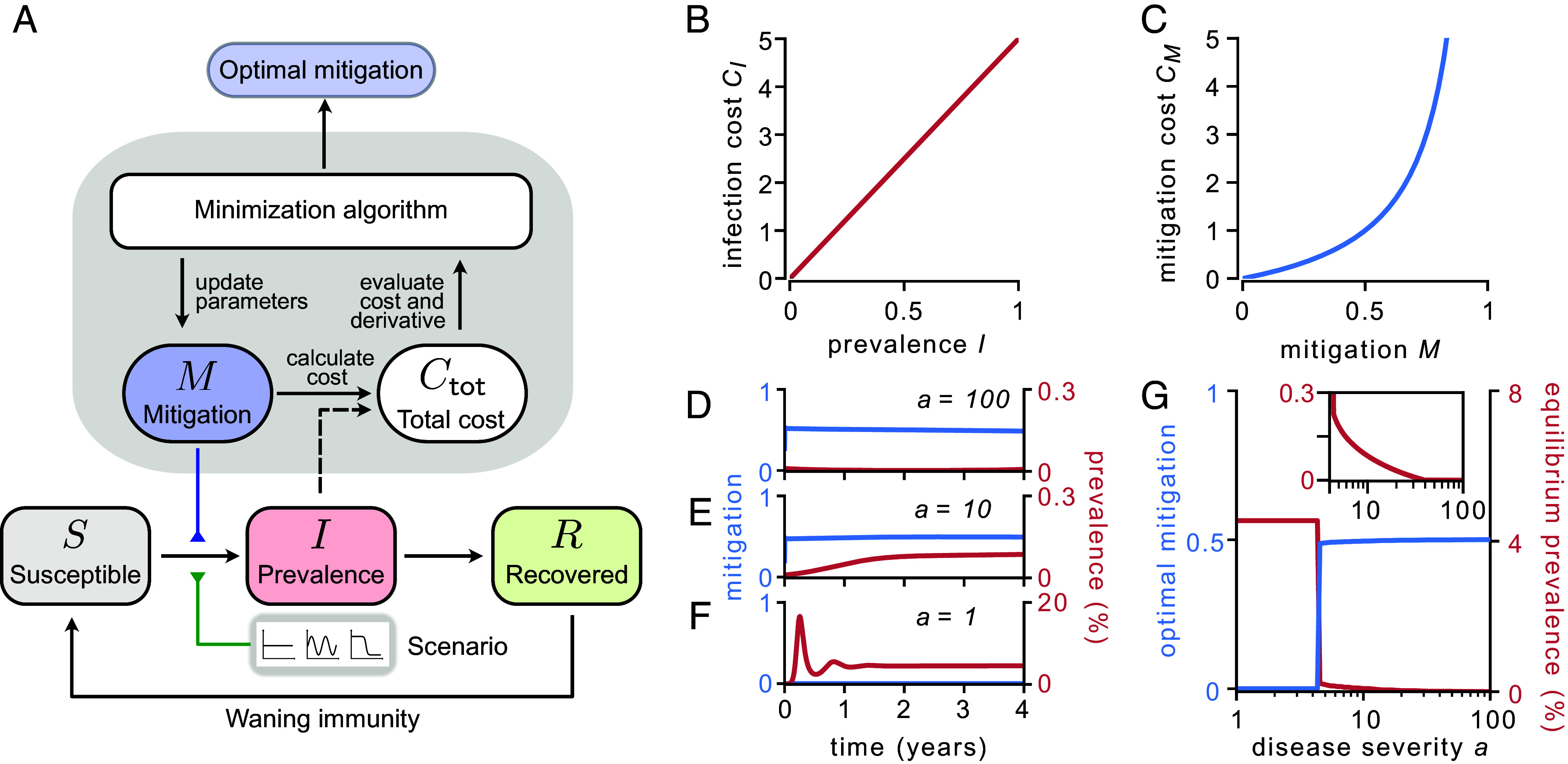
Overview of the optimization framework—For a constant reproduction number, optimal mitigation is all-or-nothing. (*A*) The SIRS model consists of three compartments: Susceptible (S), Infectious (I), and Recovered (R). The flow from Susceptible to Infectious is determined by the effective reproduction number $R_{\mathrm{eff}}$, which is dependent on the scenario (for example, seasonality) and the mitigation M. For each simulation, the total cost $C_{\text{tot}}$ is calculated. By using the total cost and its gradient, the minimization algorithm (L-BFGS) updates the mitigation parameters and eventually returns the optimal mitigation when a local minimum of the cost function is found. (*B*) We assume that the infection cost increases with the prevalence. (*C*) We assume that the mitigation cost grows supralinearly with mitigation strength. The function diverges because it is impossible to realize complete halt of transmission, i.e., when mitigation M=1. (*D*–*F*) For any disease severity a, which defines the ratio between infection and mitigation cost, optimal mitigation converges to a steady state: the optimal mitigation is high for high and intermediate disease severity (*D* and *E*), whereas the prevalence is high for low disease severity respectively (*F*). We will be mostly interested in the intermediate (a=10) regime. (*G*) The transition between the low and the high optimal mitigation regime is abrupt: at the critical disease severity $a_{\mathrm{crit}}$, mitigation jumps from 0 to approximately $M=1-1/R_{0}=0.5$, resulting in a low, approximately constant prevalence.

We assume that the infection cost increases approximately quadratically with the prevalence I up to 0.1% of the population and transitions to a linear increase beyond this threshold (*SI Appendix*, Fig. S1, *Inset*), which can be interpreted as a hospitalization threshold (Fig. 1*B*; we also consider other cost functions, see *SI Appendix*, *Supporting Information Text A.2*):

[1] $$\begin{array}{c} \begin{array}{cc} C_{I}\left(t\right)\;= & \frac{I(t)}{v}+v\left(I\left(t\right)-0.001\right)\cdot \\ & \left(1-{\left[1+\exp(1000\left(I\left(t\right)-0.001\right))\right]}^{-1}\right)+w, \end{array} \end{array}$$

where v=4.7913 and w=0.0012885 scale the transition from quadratic to linear growth of the infection cost. This pattern reflects the escalating costs associated with rising infection rates, which we assume become linear (i.e., simply proportional to the number of infectious persons) once healthcare systems reach their hospitalization capacity.

The mitigation cost follows a diverging function, where M(t)∈[0,1) represents the mitigation level (Fig. 1*C*).

[2] $$\begin{array}{c} C_{M}\left(t\right)=\frac{M\left(t\right)}{1-M\left(t\right)}. \end{array}$$

As in any rational mitigation scheme, one first uses low-cost measures (e.g., hygiene and mask recommendations). If stronger mitigation becomes necessary, costs rise supralinearly due to increasing costs when further transmission is reduced. As M approaches 1, the cost diverges, reflecting the practical impossibility of completely stopping transmission due to unavoidable transmission routes (e.g., healthcare worker–patient interactions; or noncompliance with social distancing).

The total cost, $C_{\mathrm{tot}}$, is the sum of infection and mitigation costs and is integrated over the entire simulation period (Eq. **3**). To account for the relative importance of these costs, we introduce a disease severity parameter, a, which scales the infection costs relative to mitigation costs:

[3] $$\begin{array}{c} C_{\mathrm{tot}}=\int_{0}^{T}\left(aC_{I}\left(t\right)+\;C_{M}\left(t\right)\right)dt. \end{array}$$

Because the total cost is defined only up to an arbitrary multiplicative constant, absolute cost values have no intrinsic interpretation. Instead, a encodes how strongly infections are penalized relative to mitigation, and thereby shapes the cost landscape that determines the optimal mitigation strategy. Note that a is a characteristic of a disease, but also partially subjective as it encodes normative or cultural judgments about how infection-related harms should be weighed against the societal and economic costs of mitigation.

For comparisons across scenarios, we keep the severity fixed. This ensures that the underlying trade-off between infection and mitigation costs remains unchanged, so that differences in optimal strategies can be attributed solely to epidemiological or structural variations rather than to a redefinition of the cost balance. In this sense, identical values of a correspond to the same implicit valuation of infections across scenarios. To further facilitate comparison across scenarios with differing durations or dynamics, we report costs as mean daily values. Although the costs are expressed in arbitrary units (a.u.), they provide interpretable qualitative insights. In our model, a mitigation level of M=0.5 corresponds to a cost of $C_{M}=1$ a.u.. Due to the superlinear nature of mitigation costs (Fig. 1*C*), increasing mitigation to $M=\frac{2}{3}$ already doubles the cost ($C_{M}=2$ a. u.). This implies that mitigating a disease with $R_{0}=3$ is approximately twice as costly as mitigating one with $R_{0}=2$, underscoring the need for efficient mitigation strategies.

### 1.2. Disease Dynamics.

We use an SIRS model (40), with the compartments S, I, and R representing the susceptible, infectious, and recovered fractions of the population, respectively:

[4] $$\begin{array}{c} \begin{array}{cc} \dot{S} & =-R_{\mathrm{eff}}\left(t\right)\cdot \gamma \cdot I\cdot S+\nu \left(1-S-I\right)\\ \dot{I} & =\;\;\;R_{\mathrm{eff}}\left(t\right)\cdot \gamma \cdot I\cdot S-\gamma \cdot I\\ R & =1-S-I. \end{array} \end{array}$$

$R_{\mathrm{eff}}$ is the effective reproduction number, γ the recovery rate, and ν the waning rate. The effective reproduction number, $R_{\mathrm{eff}}$, is modeled as a fractional reduction of the basic reproduction number $R_{0}$,

[5] $$\begin{array}{c} R_{\mathrm{eff}}\left(t\right)=R_{0}\left(t\right)\left(1-M(t)\right), \end{array}$$

where M(t) denotes the time-dependent mitigation, and $R_{0}$ is the basic reproduction number (which can be time-varying because of seasonality). An overview of all parameters is given in *SI Appendix*, Table S1. The initial conditions do not influence the long-term behavior of the SIRS model; regardless of their starting values, the system will eventually reach the same equilibrium or cyclical pattern. Modifications to this model to include different scenarios, like seasonality or vaccination, are described in *SI Appendix*, *Supporting Information Text A.1*.

### 1.3. Mitigation and Optimization.

The optimal mitigation strategy is determined by minimizing the total cost within a discrete-time optimal control framework based on the SIRS dynamics and cost function defined above. The mitigation M(t) acts as the control variable and is parameterized by assigning one optimization parameter to each time step ($\hat{=}$ 1 d), allowing for maximal flexibility. For a given control trajectory, the dynamical system is integrated forward in time and the corresponding cost is evaluated. Gradients of the cost with respect to all control parameters are obtained via reverse-mode automatic differentiation. This defines an iterative optimization loop in which i) the system is simulated, ii) the cost and gradients are computed, and iii) the control parameters are updated using the limited-memory Broyden–Fletcher–Goldfarb–Shanno (L-BFGS) algorithm (41), until convergence (visualized in Fig. 1*A*). As the number of parameters is on the order of a few hundred, numerical differentiation with finite differences is computationally infeasible. Instead, we employ automatic differentiation using JAX (38) and Diffrax (39) in combination with the ICoMo library (42) to integrate the system and compute gradients. This approach ensures both efficient and precise optimization of the mitigation strategy. Code is available at GitHub.

## 2. Results

### 2.1. Optimal Mitigation Undergoes a Phase Transition with Disease Severity.

In the simplest case of a pandemic with a constant basic reproduction number, $R_{0}$, the optimal mitigation strategy is, as expected, to keep an approximately steady mitigation level (Fig. 1*D*–*F*). Transient deviations may occur in the initial phase before equilibrium. Depending on disease severity a, the stringency of the mitigation leads to two qualitatively distinct outcomes. For severe diseases (e.g., a=10,100; Fig. 1 *D* and *E*), high levels of mitigation are optimal, allowing prevalence to decline to very low levels. In contrast, for mild diseases (e.g., a=1, Fig. 1*F*), the cost of mitigation outweighs its benefits, making it more cost-efficient not to implement any mitigation measures at all.

In the absence of mitigation, we observe equilibration dynamics at the beginning, particularly pronounced for mild diseases, here a=1, where mitigation is absent and multiple infection waves occur. These waves arise due to the initially naive population and are a characteristic feature of SIRS dynamics. The nature of these transient dynamics depends on the transmission rate, which in this case is effectively reduced by the mitigation factor (1−M). For mild diseases (a=1), we see full oscillatory waves before settling into equilibrium. In contrast, for more severe diseases (a=10,100), the system exhibits a smoother monotonic approach to equilibrium without pronounced waves. In all cases, however, prevalence ultimately converges to its equilibrium level.

The cleanest analytical case is a linear infection cost $C_{I}\left(I\right)=I$ together with the default mitigation cost $C_{M}\left(M\right)=M/\left(1-M\right)$. In this setting, minimizing the total cost as a function of M yields an exactly abrupt transition (*SI Appendix*, *Supporting Information Text C*): For $a<a_{\mathrm{crit}}$, the optimum is $M_{\mathrm{opt}}=0$, while for $a>a_{\mathrm{crit}}$ it jumps to $M_{\mathrm{opt}}=\tilde{M}=1-1/R_{0}$, where $\tilde{M}$ is the mitigation level that keeps $R_{\mathrm{eff}}=1$. The transition is mathematically exact: there is no interior optimum, and intermediate mitigation is strictly suboptimal.

This all-or-nothing structure is not specific to the linear infection cost or the default mitigation cost. We show in *SI Appendix*, *Supporting Information Texts D and E* that the same binary transition holds for any infection cost of the form $C_{I}\left(I\right)=I^{\alpha }$ with α≤1, and any mitigation cost $C_{M}$ such that $C_{M}^{\prime }\left(M\right){\left(1-M\right)}^{2}$ is nonincreasing on $\left[0,\tilde{M}\right]$. The latter condition requires $C_{M}$ to grow at least as fast as M/(1−M); for the family $C_{M}\left(M\right)=M/\left(1-M^{\zeta }\right)$, it reduces to ζ≥1. Whenever both conditions hold, the optimum lies at one of the boundary points $\left\{0,\tilde{M}\right\}$, and the critical severity generalizes to

[6] $$\begin{array}{c} a_{\mathrm{crit}}=\frac{C_{M}\left(\tilde{M}\right)\;{\left(\gamma +\nu \right)}^{\alpha }\;R_{0}^{\alpha }}{\nu^{\alpha }\;{\left(R_{0}-1\right)}^{\alpha }}. \end{array}$$

Different cost choices therefore shift the value of $a_{\mathrm{crit}}$ through the factor $C_{M}\left(\tilde{M}\right)$, but do not alter the binary structure of the optimal strategy. For our default cost functions, $C_{M}\left(\tilde{M}\right)=R_{0}-1$, and the formula reduces to $a_{\mathrm{crit}}=\left(\gamma +\nu \right)R_{0}/\nu$, which evaluates to 4.4 for the parameters of Fig. 1. Outside the regime of the theorem, that is, for superlinear infection cost (α>1) or for a mitigation cost that rises too fast at low M (ζ<1 in the family $C_{M}\left(M\right)=M/\left(1-M^{\zeta }\right)$), the proof no longer applies. Numerical exploration confirms that the all-or-nothing structure can break down in theses regimes: for example, for (α,ζ)=(2,0.1), the optimal mitigation varies smoothly with disease severity rather than jumping abruptly (*SI Appendix*, Fig. S6).

The infection cost used throughout this paper has a kink at the hospitalization threshold $I_{h}\approx 0.1\%$ (Eq. **1**), placing it outside the regime covered by the theorem above. A natural question is whether this kink can sustain an interior optimum at or near $I_{h}$, where the marginal infection cost is lowest. To address this, we analyze a bilinear simplification of $C_{I}$ with slope 1 below $I_{h}$ and slope 5 above (*SI Appendix*, *Supporting Information Text G*). The kink at $I_{h}$ introduces a third candidate optimum, $M_{h}$, which pins the equilibrium prevalence exactly at $I_{h}$. Comparing C(0), $C(M_{h})$, and $C(\tilde{M})$ identifies three regimes in disease severity: for low a, no mitigation is optimal ($M_{\mathrm{opt}}=0$); for intermediate a, the optimum pins prevalence at the threshold ($M_{\mathrm{opt}}=M_{h}$, $I_{\mathrm{opt}}^{*}=I_{h}$); for high a, full suppression at $\tilde{M}$ takes over (visible in *SI Appendix*, Fig. S3). The smooth infection cost used in the main text regularizes this picture: the discrete jump in $C_{I}^{\prime }$ at $I_{h}$ becomes a smooth crossover, and the lock at $I_{h}$ becomes a smooth decrease of $I_{\mathrm{opt}}^{*}$ toward zero. This produces the small shoulder visible in the *Inset* of Fig. 1*G*, where the equilibrium prevalence decreases gradually from approx. 0.3% to zero, rather than jumping discontinuously. The all-or-nothing structure is therefore not violated but softened in a structured, predictable way by the kink in $C_{I}$.

We note that, even though the mitigation cost is strictly convex, the abrupt switch between the two regimes resembles bang-bang-type behavior, which would be expected for linear costs. However, unlike classical bang–bang control, where the control switches between bounds over time along a single optimal trajectory, the transition here arises primarily as the severity, meaning the ratio between infection and mitigation costs, varies across disease scenarios, reflecting a threshold-type shift between mitigation regimes rather than a temporal switching policy. This shift arises due to the nonlinear dependence of the epidemic dynamics on the mitigation.

Together, these results sharpen the all-or-nothing picture into a conditional principle: for long-term SIRS dynamics with cost functions in the regime of the theorem, the optimal mitigation is binary, taking the values 0 or $\tilde{M}=1-1/R_{0}$ depending on disease severity. Outside this regime, the kink in $C_{I}$ at $I_{h}$ can sustain an intermediate pinning regime, and sufficiently soft mitigation costs combined with superlinear infection costs can wash out the transition entirely.

In the following analyses, we focus on the more relevant case of severe diseases requiring mitigation, and fix the severity parameter at a=10 unless stated otherwise.

### 2.2. Optimal Mitigation Alters Seasonal Outbreak Severity and Timing.

**Fig. 2.**
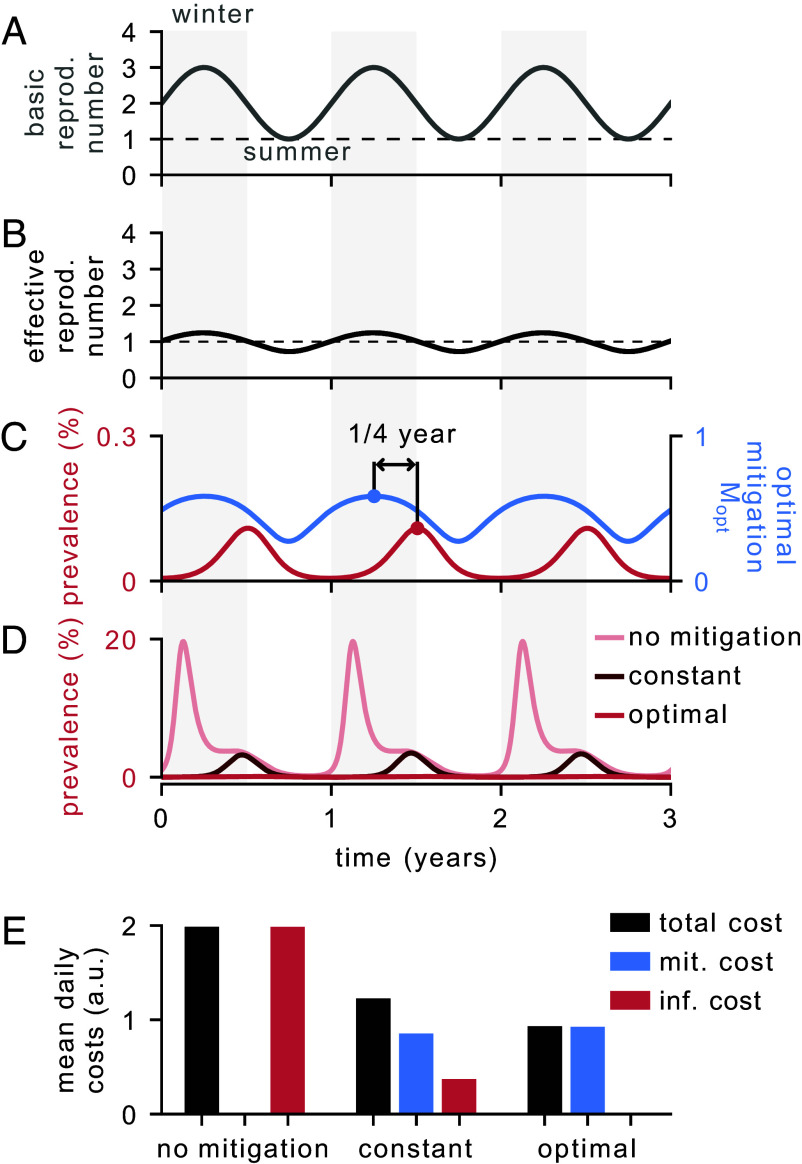
Seasonally timed mitigation delays and flattens infection waves. (*A*) We assume a sinusoidal time evolution of the basic reproduction number, representing the seasonality of disease transmission (maximal in winter, minimal in summer). (*B*) With an optimal mitigation strategy, the resulting effective reproduction number ($R_{\mathrm{eff}}\left(t\right)=R_{0}\left(t\right)\left(1-M(t)\right)$) has a lower amplitude than the basic reproduction number and oscillates around 1. (*C*) The peak of the resulting prevalence is shifted by π/2 (1/4 y) compared to the peak of the optimal mitigation. (*D*) Compared to scenarios with no mitigation or a comparable constant mitigation, the prevalence of the optimal strategy is significantly lower. (*E*) In the case of no mitigation, the mean daily costs, which are only comprised of infection costs, are approximately double those of the optimal scenario. In the constant scenario, mitigation costs are similar to those in the optimal scenario, however, infection costs are notably higher, leading to higher total costs. Results for different disease severities, cost functions, mean values, and amplitudes of the basic reproduction number can be found in *SI Appendix*, Figs. S7–S10.

Many infectious diseases, particularly respiratory infections, exhibit seasonal patterns in incidence. These patterns are often linked to fluctuations in the basic reproduction number, with transmission typically increasing during winter and decreasing in summer, as observed for Influenza virus, human coronavirus (HCoV), and human respiratory syncytial virus (RSV) (43). Investigating these dynamics is important for understanding whether optimal control strategies should be reactive or preventive in nature, as the time-dependent reproduction number, in combination with nonlinear long-term effects of mitigation, may lead to nontrivial outcomes. We implement these seasonal fluctuations using a sinusoidal basic reproduction number with a period of one year (Fig. 2*A* and *SI Appendix*, Eq. **2**).

In the presence of seasonal fluctuations, the optimal mitigation also follows an approximately sinusoidal pattern, oscillating in phase with the basic reproduction number (Fig. 2*C*). The mitigation measures thereby counteract most of the seasonal variation in transmission: the resulting effective reproduction number (Eq. **5**) is still periodic (Fig. 2*B*), but oscillates around 1 with a reduced amplitude (±0.26 instead of ±1).

This qualitative behavior is robust: for a wide range of parameters and different cost functions, and a range of seasonality shapes, the optimal mitigation tracks the basic reproduction number and maintains the effective reproduction number close to one (*SI Appendix*, Figs. S5 and S7–S10). The exact mitigation strategy depends on the cost function: for example, for a logarithmic mitigation cost (*SI Appendix*, Eq. **13**), the optimal mitigation oscillates in such a way that the effective reproduction number remains approximately constant at 1 throughout the whole year (*SI Appendix*, Fig. S7). For other cost functions (Eq. **2** and *SI Appendix*, Eq. **14**) the effective reproduction number oscillates around 1 as the optimal mitigation is not able to completely balance the basic reproduction number under these costs. For mild diseases, mitigation is absent and infection dynamics are primarily controlled by immunity (*SI Appendix*, Fig. S8).

Interestingly, despite both the basic and effective reproduction numbers being maximal in winter, the peak of the prevalence is in spring (Fig. 2*C*). Prevalence increases for half of the year from mid-fall to mid-spring, where the effective reproduction number exceeds 1. For the other half of the year, prevalence decreases. Consequently, it peaks in spring, where the effective reproduction number crosses 1. Between the peaks of mitigation (winter) and prevalence (spring), there is a shift of exactly three months, or π/2. This shift of π/2 is independent of the period of the fluctuations (*SI Appendix*, Fig. S5). Surprisingly, the same three-months shift also holds for less symmetrical seasonal patterns.

To evaluate the benefits of optimal mitigation, we compare it to two alternative strategies: no mitigation (M(t)=0) and constant mitigation set to the mean value of the optimal strategy ($M\left(t\right)=\bar{M_{\mathrm{opt}}\left(t\right)}$) such that the overall mitigation cost is comparable (Fig. 2 *D* and *E*). In the absence of mitigation, a large infection wave occurs in winter, followed by a period of sustained prevalence in spring (Fig. 2*D*). Under constant mitigation (with the same mean value of mitigation as in the optimal case), a single but lower wave peaks in early spring (reduced by 82%), reducing the total cost by 38% compared to no mitigation (Fig. 2*E*). Optimal mitigation also results in a single peak in prevalence in spring; however, its height is considerably lower—by 99% compared to no mitigation and by 97% compared to constant mitigation—rendering it barely visible in Fig. 2*D*. The optimal total cost decreases by 53% compared to the no mitigation scenario and by 24% compared to the constant mitigation, with infection costs nearly absent (Fig. 2*E*). This comparison highlights a key policy insight: not only mean mitigation but also its precise temporal profile matters; even with identical mean mitigation and nearly identical mitigation costs, the total costs and infection costs can differ substantially.

In summary, optimal mitigation in the presence of seasonality not only reduces the total costs and outbreak size but also shifts the seasonal peak from winter to spring (in the model precisely by π/2). The reduction in total costs underscores the value of proactive mitigation strategies implemented ahead of anticipated waves.

### 2.3. Vaccination Allows for a Decrease in Mitigation Measures.

Vaccines, when they become available, play a crucial role in disease management. They decrease disease transmission within a population for a sustained period. Even if vaccination does not guarantee complete immunity, people are less likely to get infected or infect others if they are vaccinated (44, 45).

To include the protective effect of vaccination in the SIRS model, we split the S, I and R compartments into two compartments each, one for vaccinated and one for unvaccinated people (*SI Appendix*, Eqs. **4**–**9**). Vaccinated individuals are assumed to be less likely to become infected and to transmit the disease. Their contribution to transmission is reduced by a factor η, which captures the vaccine’s effectiveness in lowering overall transmission. Accordingly, we define an adjusted effective reproduction number, $R_{\mathrm{vac}}$, for vaccinated individuals.

$$\begin{array}{c} R_{\mathrm{vac}}=R_{\mathrm{eff}}\left(1-\eta \right). \end{array}$$

**Fig. 3.**
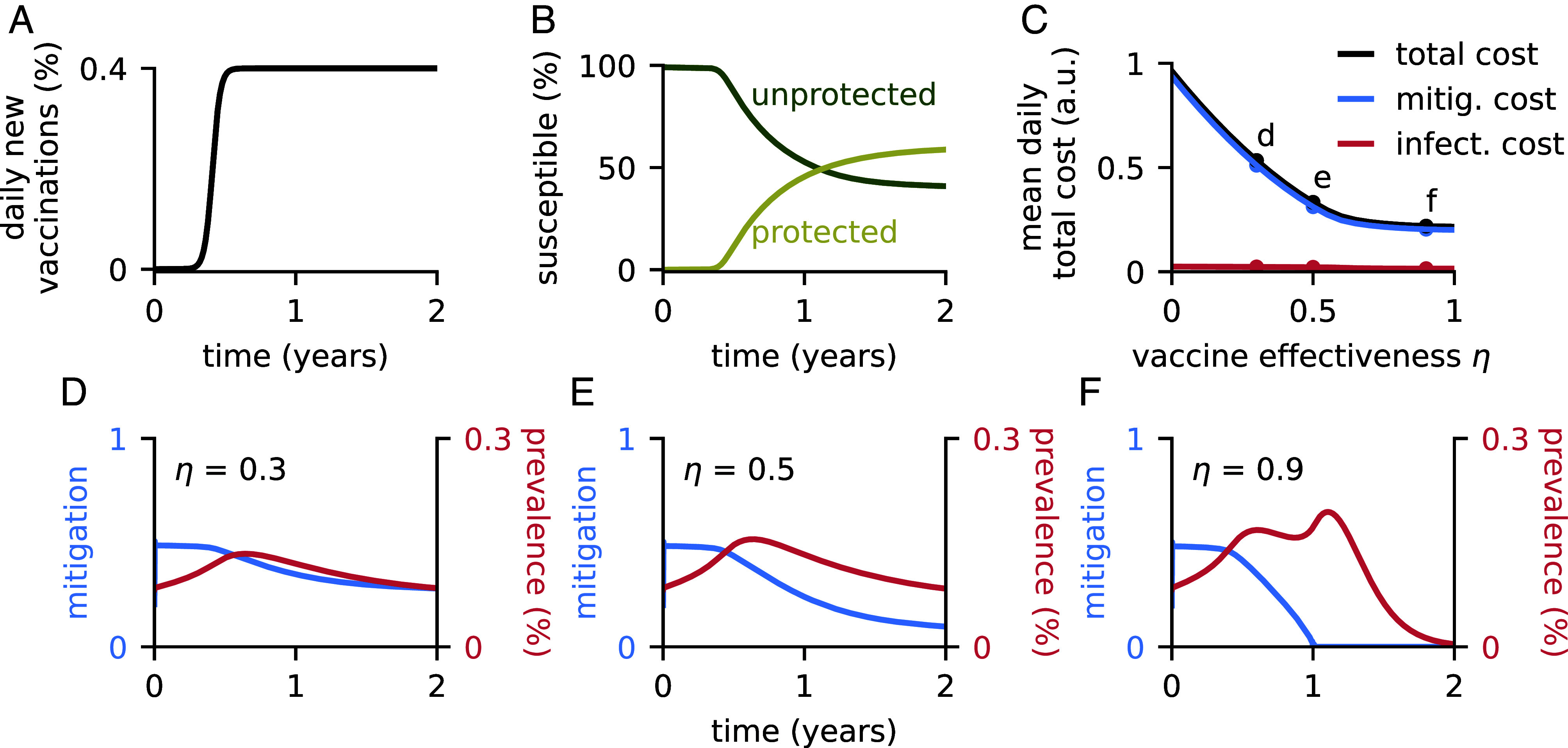
Impact of vaccine rollout on optimal mitigation and prevalence patterns. (*A*) We assume a rapid increase of the daily new vaccinations at the beginning of the vaccination campaign before they plateau. This steady distribution mainly represents booster vaccinations keeping a part of the population partially immune (*SI Appendix*, Eq. **3**). (*B*) The whole population will not reach the vaccinated (protected) status as we assume a waning vaccine immunity ($\nu_{\mathrm{vac}}=1/150$ d^−1^, see *SI Appendix*, Eqs. **4** and **5**). (*C*) The mean daily total costs decrease with increasing vaccine effectiveness up to the point where the vaccine is effective enough to eradicate the disease on its own. The values for the example plots in panels *D*–*F* are marked with dots. The cost is integrated over the total simulation length (3 y). (*D*–*F*) Vaccination enables a reduction in mitigation stringency and, with high vaccine effectiveness, may even allow for a complete halt (*F*). During this period, however, one or more small infection waves may still occur. Results for different disease severities and cost functions can be found in *SI Appendix*, Figs. S11 and S12.

Each day, a fixed number of individuals move from the unvaccinated susceptible compartment to the vaccinated susceptible compartment (Fig. 3 *A* and *B* and *SI Appendix*, Eq. **3**). Immunity from vaccination wanes at rate $\nu_{\mathrm{vac}}$, returning individuals to the unvaccinated susceptible pool. As starting condition, we assume that the vaccination campaign only starts after a longer time, i.e., after the system has reached its endemic equilibrium under optimal mitigation.

The increasing immunity due to vaccination enables a gradual reduction in other mitigation measures (Fig. 3*D*–*F*). Before the vaccine rollout begins, the optimal mitigation strategy is approximately constant, similar to the baseline scenario without vaccination (Fig. 1*D*–*F*). As vaccination progresses, the number of unvaccinated susceptible people declines (Fig. 3*B*). The extent to which mitigation then decreases depends on the vaccine effectiveness η. For sufficiently high vaccine effectiveness, mitigation measures can even be stopped entirely (Fig. 3*F*). Interestingly, even under optimal control, small infection waves may occur during the early stages of vaccine rollout, before the population reaches a new equilibrium (Fig. 3*D*–*F*). This highlights that optimal mitigation strategies during vaccination campaigns may involve nontrivial time dynamics and can yield counterintuitive patterns in infection prevalence.

The resulting total cost decreases nonlinearly with increasing vaccine effectiveness (Fig. 3*C*). A vaccine effectiveness of η=0.5 already reduces the total cost by 65% compared to the scenario without vaccination. Overall, effective vaccination reduces the need for other measures, leading to substantial economic savings and greater personal freedom.

We note that in real-world scenarios such as the COVID-19 pandemic, vaccination not only reduced transmission, but also significantly lowered disease severity in breakthrough infections (46). In our framework, such effects could be captured by allowing the severity parameter a to decrease with cumulative vaccination coverage. Note that we do not include vaccination costs in our model since the vaccination rate is independent of the mitigation strategy and we do not aim to optimize vaccine distribution. However, this framework could be expanded to include the cost of the vaccination rate, hence obtaining an estimation of the most cost-effective timing of a vaccination campaign.

### 2.4. Delaying Mitigation Measures Entails Additional Costs.

**Fig. 4.**
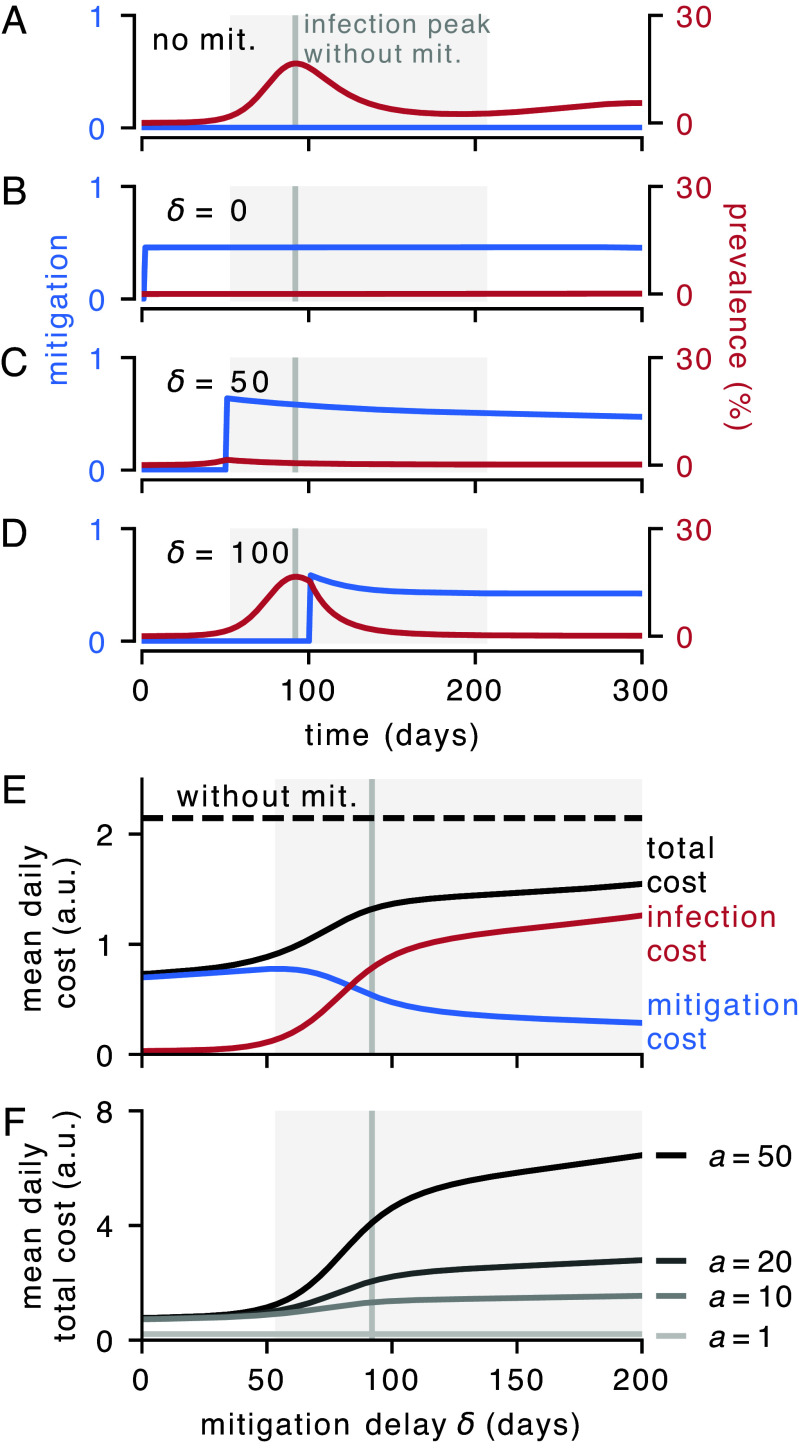
Delaying mitigation increases total cost—especially for more severe diseases. (*A*–*D*) Delaying mitigation for (*C*) 50 or (*D*) 100 d significantly increases the total prevalence and requires temporary stronger mitigation to control case numbers, which are in all cases lower than without mitigation (*A*). The gray line and area mark the peak and interval of the first infection wave in the case of no mitigation. (*E*) Total cost increases with delay, driven by higher infection costs—even though mitigation becomes shorter and cheaper. The total simulation length, over which the costs are calculated, is 455 d. (*F*) The change in total costs by delaying mitigation, i.e., the “cost of waiting,” increases the more severe the disease is. Results for different disease severities and cost functions can be found in *SI Appendix*, Figs. S13 and S14.

It is known that delayed onset of mitigation in the case of a new pandemic can incur high additional costs (7, 47, 48). Here, we quantify these costs by modeling scenarios in which mitigation is initially absent and subsequently applied optimally after a fixed delay δ, measured from day zero, with $I\left(0\right)=10^{-4}$ (approx. 8,000 individuals in Germany), a plausible early prevalence for a country that is not the initial epicenter, given the potential for rapid undetected spread (as observed, for example, in COVID-19). Specifically, we set M(t)=0 for t<δ and optimize mitigation for t≥δ (Fig. 4). For our standard disease severity (a=10), mitigation begins with a sharp increase in intensity and then slowly converges to its equilibrium value, consistent with the nondelayed results (Fig. 1*D*–*F*). In addition, we explore how the results depend on the choice of cost function and find qualitatively similar behavior across variations (*SI Appendix*, Fig. S13).

Prior to the onset of mitigation (t<δ), the system follows standard SIRS dynamics (Fig. 4*A*–*D*). The size of the resulting initial infection wave increases with the delay, driving up the total cost (Fig. 4 *E* and *F*). While the infection cost consistently increases, the mitigation cost decreases with δ (apart from a small initial rise) but does not offset the increasing infection cost. The initial rise of the mitigation cost reflects a catch-up effect: the system must mitigate more aggressively once intervention begins in order to counteract an ongoing infection wave. Consequently, total cost increases monotonically with delay, though it always remains below the cost of an uncontrolled epidemic. This cost increase becomes more pronounced as disease severity a rises (Fig. 4*F*). This finding underscores that even late intervention is still beneficial.

For interventions that start after the peak of the initial infection wave (gray vertical line in Fig. 4), the total cost increases at a slower rate. This suggests that rapid response is most critical before the first infection peak would occur, as early intervention reduces the prevalence and, consequentially, the total costs of the outbreak (Fig. 4*E*).

To contextualize the cost of delay, consider the case of a=10. Even a short delay of δ=7 d results in 0.39% more of the population getting infected during the simulation compared to immediate intervention, i.e., δ=0 (5.55% vs. 5.16%). In a country like Germany, this translates to roughly 300,000 additional infections—highlighting the real-world consequences of even modest delays in implementing mitigation measures.

Importantly, our framework also allows to consider the total cost, though in arbitrary units. For the example above, waiting 7 d increases the total cost by 1.18%, compared to an increase in infections of 7.56%. This demonstrates the difficulty in assessing the quality of mitigation strategies using infection numbers alone.

## 3. Discussion

We developed a computationally efficient framework that combines optimal control with simulation of disease dynamics to optimize time-dependent mitigation strategies for pandemics and epidemics. Its versatility allows for adaptation to various infectious diseases, scenarios, and policy objectives by modifying the underlying disease model, parameters, and cost functions. For example, incorporating seasonal fluctuations required only minor modifications to the base SIRS model. This makes the framework well-suited to determine effective mitigation strategies during future disease outbreaks. We applied the framework to diverse scenarios, including seasonality, vaccination rollout, and delayed intervention, showing several unexpected results.

One of the most striking findings is that optimal mitigation exhibits a sharp transition depending on disease severity, which scales the infection cost relative to the mitigation cost. For mild diseases, the cost of intervention outweighs its benefits, and the optimal strategy is to implement no mitigation at all. For severe diseases, the optimal strategy abruptly switches to mitigation at a level $M=1-\frac{1}{R_{0}}$, thereby maintaining $R_{\text{eff}}=1$. Within a broad regime of cost-function shapes that we characterize analytically, this transition is exact: there is no stable intermediate regime.

Both the critical disease severity and the optimal mitigation level depend on the disease parameters; they increase with $R_{0}$. Different diseases, characterized by different basic transmission rates $\beta_{0}$, recovery rates γ, and waning immunity rates ν, therefore change the critical disease severity or mitigation stringency needed, but do not affect the existence of a critical threshold. Only in the case of $R_{0}<1$ does the disease die out by itself, requiring no mitigation. Beyond the disease parameters, the all-or-nothing structure also depends on the shape of the cost functions: it holds whenever the infection cost grows no faster than linearly in prevalence and the mitigation cost satisfies a technical convexity condition that covers a broad family of plausible cost shapes (in particular, all costs $C_{M}\left(M\right)=M/\left(1-M^{\zeta }\right)$ with ζ≥1). Outside this regime, in particular when the infection cost is strongly superlinear and the mitigation cost rises too quickly at low M, the transition can smooth out and intermediate mitigation levels become optimal. The all-or-nothing behavior should therefore be understood as a structural principle that holds across a wide but not unlimited class of cost models, rather than as a universal property of disease mitigation.

The existence of such a sharp transition is consistent with previous studies (19, 22, 27, 28, 36). However, the underlying drivers differ. Prior works consider epidemics without reinfection, meaning the disease eventually dies out, where transitions depend on altruism and initial infections (27), the cost of social distancing and epidemic duration (36), value of life (19, 22), or population size, infection cost, and optimization time (28). In contrast, we assume long-term epidemics including reinfection, and the transition emerges as a function of disease severity, thereby arising endogenously from disease characteristics rather than being driven by external parameters such as the time horizon. Despite these differences, our results are in qualitative agreement with several of the other studies in that the transition reflects a trade-off between competing costs, such as costs of infections vs. control efforts (28, 36), or mortality vs. the economic costs (19, 22). Other studies that included reinfection in their models optimized both mitigation and treatment. These kinds of dynamics are qualitatively equivalent to ours. However, the authors did not show explicitly a transition of one global minimum to another (20, 23).

Another important finding is that if seasonal fluctuations in the basic reproduction number are introduced, optimal mitigation is in phase with the seasonality across a wide range of seasonality shapes (*SI Appendix*, Fig. S5), and the resulting prevalence peak lags behind by exactly a quarter of a period. This is also to be expected from our analytical calculations which showed that the mitigation threshold, which determines the growth–decay boundary, $M_{\text{thresh}}\left(t\right)=1-\frac{1}{R_{0}\left(t\right)\;S\left(t\right)}$, depends on $R_{0}\left(t\right)$ (*SI Appendix*, *Supporting Information Text B*). This finding is consistent with other results (14), which showed that adjusting mitigation to maintain $R_{\text{eff}}\approx 1$ is optimal in settings with a time-varying basic reproduction number. As a result, the infection peak is moved from fall/winter to spring, when the probability of getting infected is naturally lower. This contrasts with real-world responses during COVID-19, where interventions were often implemented in response to current prevalence rather than anticipating future risk. Even though some countries adopted proactive mitigation strategies, most took a reactive approach (49). Such reactive strategies can be explained by communication challenges and behavioral fatigue, which complicate the implementation of preemptive measures (50–53). However, our framework provides a basis for communicating why interventions implemented before visible surges are not only justified but often more effective. Furthermore, given that many infectious diseases exhibit consistent seasonal trends, these patterns could be used to anticipate periods of increasing transmission and guide timely, proactive mitigation efforts.

During vaccination rollout, the optimal strategy involves gradually reducing mitigation as population-level immunity builds. This aligns with previous studies (14, 21, 37, 54). However, we also observed that small infection waves may still occur even under optimal control. This underscores the fact that the relationship between vaccination, mitigation, and disease prevalence is dynamically complex and not necessarily monotonic. Moreover, it suggests that infection dynamics alone are an unreliable indicator of the optimality of mitigation strategies.

At the onset of a new outbreak, delayed mitigation is known to incur substantial costs (47, 55). A key strength of our framework is that it allows us to quantify this “cost of waiting” precisely. We find that infection costs increase with longer delay, while mitigation costs may transiently rise as the system “catches up” to suppress an outbreak, before eventually declining. Quantitatively, the benefit of acting early is highest before the first infection peak would occur, preventing the high peak; and the more severe the disease, the higher the difference between acting earlier or later. Other studies similarly highlight the importance of early implementation and the increased stringency required when measures are delayed (15, 25, 33).

While the optimal policy is often observed to approximately track $R_{\mathrm{eff}}=1$, this behavior is not imposed or enforced within the optimization problem, but instead emerges endogenously from the optimal solution. In the SIRS setting, waning immunity induces a continual replenishment of susceptible individuals, which introduces a strong intertemporal coupling in the control problem. Consequently, mitigation decisions affect not only current infection levels but also the future susceptibility landscape and thus future transmission potential. This makes it plausible that optimal strategies could involve boundary solutions or oscillatory behavior rather than maintaining a reproduction number near unity. The fact that the optimal solution nevertheless organizes around $R_{\mathrm{eff}}=1$, both for constant $R_{0}$ (where we prove it analytically for a broad class of cost functions, see *SI Appendix*, *Supporting Information Texts B–E*) and across a wide range of seasonality shapes, is therefore a nontrivial outcome of the optimization.

The optimal mitigation stringency for severe diseases depends on disease characteristics like the basic reproduction number $R_{0}$. In real life, it is hard to estimate the exact value of such parameters and track their temporal changes. This complicates the practical realization of the optimal measures. Consequently, strategies may be off, for example due to delayed or incomplete information, and communication challenges. This entails increased costs as well as possibly higher prevalence. Additionally, depending on the cost function, keeping $R_{\mathrm{eff}}=1$ may not always be the most cost-efficient solution, as we found, e.g., for the seasonality, where for some cost functions the optimal strategy is unable to completely counteract the changing transmission rate so that we still see annual infection waves.

The choices of cost functions are informed by general assumptions about the dynamics of disease control. For mitigation, we assume that costs grow superlinearly with intensity, based on the idea that low-cost measures are implemented first, and later, stricter interventions (e.g., lockdowns) incur successively higher costs. Any rational mitigation strategy follows such a superlinear cost function. This assumption also reflects that instantaneous suppression (i.e., $R_{\mathrm{eff}}=0$) is practically impossible, due to essential interactions and imperfect compliance. Within a broad family of cost shapes, the precise choice of the cost function does not impact the results qualitatively (*SI Appendix*, Figs. S7, S11, and S13); we make this regime precise in *SI Appendix*, *Supporting Information Text D*. A limitation of our mitigation cost function is that it is independent of the current prevalence, whereas in real life mitigation is typically cheaper when case numbers are low, e.g., because of test-trace-and-isolate strategies (7). Nevertheless, we believe that our choice of a prevalence-independent mitigation cost function is reasonable and general.

Similarly, we model infection costs as increasing superlinearly with prevalence up to the threshold I≈0.1%, reflecting reduced quality of care as staff and resources become constrained, which in turn raises treatment costs, for example through longer recovery times and long-term complications. Beyond hospital capacity, additional patients receive similarly limited care, so the marginal cost per infection is roughly constant, justifying the linear continuation of the cost function. While exact cost curves are hard to quantify empirically, our assumptions are grounded in general behavioral and systemic considerations, and the qualitative results we report remain consistent under different functional forms within the regime characterized in *SI Appendix*, *Supporting Information Text E* (*SI Appendix*, *Supporting Information Text H*). We note that the precise value of the hospitalization threshold does not qualitatively affect the regime structure of the optimal solution (*SI Appendix*, Fig. S3). For mild diseases, where we find that the optimal strategy is no mitigation, infections go up to high numbers that exceed the assumed hospitalization threshold in our infection cost function. However, in case of a mild disease, the hospitalization rate is low, so the assumed threshold does not hold. One could implement this by making the hospitalization threshold dependent on a. In our analysis, however, we decided to keep the hospitalization rate fixed to ensure comparability across scenarios.

The disease severity parameter a determines the ratio between infection and mitigation cost. It captures two distinct aspects. First, it reflects the intrinsic severity of the disease, such as the risk of hospitalization, long-term complications, or death. Second, it encodes normative or cultural judgments about how infection-related harms should be weighed against the societal and economic costs of mitigation. This includes, for example, how a population values the prevention of illness or loss of life relative to personal freedom, mental health, or economic stability. Consequently, its value may vary across populations, political settings, and stages of a pandemic. Rather than fixating on a single value, our contribution is to characterize how optimal strategies vary with disease severity and to identify structural principles that hold across wide parameter ranges. It then remains a task for society and politics to understand whether a specific new disease is “mild” (low a) or “severe,” and whether mitigation is warranted.

In our scenarios, we chose a disease severity of a=10 as the default value. This represents a moderately severe disease: it places the system in the mitigation-dominated regime and leads to infection levels that remain below the infection threshold I≈0.1%, or 100 per 100,000 people; Fig. 1), which reflects approximately the hospitalization capacity for COVID-19 in many countries. Hence, effectively, a severity of a=10 represents an infectious disease like COVID-19 before immunization.

There are several extensions one could implement to make the model more realistic. While we showed that the optimal mitigation strategy is robust across a range of seasonality shapes, real-world seasonal patterns are often more irregular and differ across years, which could lead to nonoptimal mitigation if society makes wrong assumptions about timing and amplitude. More broadly, the basic reproduction number is subject to fluctuations that may be only partially predictable (56), whereas our study assumes perfect knowledge and deterministic dynamics; simulating such scenarios would require techniques like model predictive control (24, 57). Beyond these dynamical considerations, several structural extensions could further enrich the framework. Incorporating heterogeneity in age, risk, or contact structure could modify the sharpness of the all-or-nothing transition, or confine it to specific subpopulations. On the economic side, our instantaneous mitigation cost does not capture path-dependent effects such as workforce exit and economic scarring, which could favor shorter and sharper interventions over sustained moderate ones; conversely, for sufficiently severe diseases, infected individuals are themselves removed from the productive workforce, introducing a nonlinear dependence of the cost of infection on severity parameter a.

The mitigation variable in our model does not correspond to a specific intervention, but rather to its overall effect on transmission. This abstraction allows our results to be interpreted flexibly: different combinations of measures (e.g., mask use, social distancing, ventilation) can be used to achieve a given mitigation level. We also do not define whether the intervention is mandatory or voluntary. The key insight is that optimal mitigation often counteracts the basic reproduction number, rather than reacting to current prevalence; this is a principle that can guide the design of proactive and cost-efficient responses in practice. Thus, although our findings are not intended as direct prescriptive policy, they provide important strategic insights into rational epidemic management.

Finally, our framework allows users to easily modify the underlying functions with minimal effort or technical expertise. To illustrate this flexibility, we provide a publicly accessible Google Colab notebook showcasing one scenario from our analysis at https://colab.research.google.com/drive/16J2hNb519R0QZyRJm_egVJGH-J5Lbjfh?usp=sharing.

In summary, we introduced a general framework for determining optimal mitigation strategies under dynamic epidemic conditions. We showed that for mild diseases, it is optimal not to intervene, whereas for severe diseases, mitigation should be adjusted dynamically to maintain $R_{\text{eff}}=1$. This enables us to maintain low levels of prevalence, thus preserving healthcare capacities, reducing deaths, and limiting societal disruption (58). These findings underscore the need for continuous monitoring of transmission dynamics and the timely adaptation of mitigation strategies.

## 4. Disclosure of Delegation to Generative AI

The authors declare the use of generative AI in the research and writing process. According to the GAIDeT taxonomy (2025), the following tasks were delegated to GAI tools under full human supervision: literature search and systematization; Proofreading and editing. GAI tool used was: https://elicit.com, Claude Opus 4.7, GPT-5.3. Responsibility for the final manuscript lies entirely with the authors. GAI tools are not listed as authors and do not bear responsibility for the final outcomes.

## Supplementary Material

Appendix 01 (PDF)

## Data Availability

All results can be reproduced in full using our code. We provide the code for the general framework., scenarios, and generating graphics at GitHub (https://github.com/Priesemann-Group/optimizing_pandemic_mitigation) (59).

## References

[1] P. Deb, D. Furceri, J. D. Ostry, N. Tawk, The economic effects of COVID-19 containment measures. Open Econ. Rev. **33**, 1–32 (2022).

[2] B. Marroquín, V. Vine, R. Morgan, Mental health during the COVID-19 pandemic: Effects of stay-at-home policies, social distancing behavior, and social resources. Psychiatry Res. **293**, 113419 (2020). 32861098 10.1016/j.psychres.2020.113419 PMC7439968

[3] I. M. Longini, E. Ackerman, L. R. Elveback, An optimization model for influenza A epidemics. Math. Biosci. **38**, 141–157 (1978).

[4] J. M. Lopes , Optimization methods for large-scale vaccine supply chains: A rapid review. Ann. Oper. Res. **316**, 699–721 (2022). 35531563 10.1007/s10479-022-04720-5 PMC9059697

[5] L. Matrajt , Optimizing vaccine allocation for COVID-19 vaccines shows the potential role of single-dose vaccination. Nat. Commun. **12**, 3449 (2021). 34103510 10.1038/s41467-021-23761-1 PMC8187351

[6] S. Han , Time-varying optimization of COVID-19 vaccine prioritization in the context of limited vaccination capacity. Nat. Commun. **12**, 4673 (2021). 34344871 10.1038/s41467-021-24872-5 PMC8333090

[7] S. Contreras , Low case numbers enable long-term stable pandemic control without lockdowns. Sci. Adv. **7**, eabg2243 (2021). 34623913 10.1126/sciadv.abg2243 PMC8500516

[8] J. M. Brauner , Inferring the effectiveness of government interventions against COVID-19. Science **371**, eabd9338 (2021). 33323424 10.1126/science.abd9338 PMC7877495

[9] T. Oraby , Modeling the effect of lockdown timing as a COVID-19 control measure in countries with differing social contacts. Sci. Rep. **11**, 3354 (2021). 33558571 10.1038/s41598-021-82873-2 PMC7870675

[10] V. Gemmetto, A. Barrat, C. Cattuto, Mitigation of infectious disease at school: Targeted class closure vs. school closure. BMC Infect. Dis. **14**, 1–10 (2014). 25595123 10.1186/s12879-014-0695-9 PMC4297433

[11] J. Dehning , Inferring change points in the spread of COVID-19 reveals the effectiveness of interventions. Science **369**, eabb9789 (2020). 32414780 10.1126/science.abb9789 PMC7239331

[12] N. Gavish, G. Katriel, Optimal regulation in a periodic environment: Insights from a simple model. *Appl. Math Optim*. **93**, 22 (2026).

[13] D. Greenhalgh, Some results on optimal control applied to epidemics. Math. Biosci. **88**, 125–158 (1988).

[14] A. Shirin, Y. T. Lin, F. Sorrentino, Data-driven optimized control of the COVID-19 epidemics. Sci. Rep. **11**, 6525 (2021). 33753777 10.1038/s41598-021-85496-9 PMC7985510

[15] G. Albi, L. Pareschi, M. Zanella, Control with uncertain data of socially structured compartmental epidemic models. J. Math. Biol. **82**, 63 (2021). 34023964 10.1007/s00285-021-01617-y PMC8141280

[16] J. M. Macalisang, M. L. Caay, J. P. Arcede, R. L. Caga-anan, Optimal control for a COVID-19 model accounting for symptomatic and asymptomatic. Comput. Math. Biophys. **8**, 168–179 (2020).

[17] P. Godara, S. Herminghaus, K. M. Heidemann, A control theory approach to optimal pandemic mitigation. PLoS ONE **16**, e0247445 (2021). 33606802 10.1371/journal.pone.0247445 PMC7894916

[18] B. R. Rowthorn, F. Toxvaerd, “The optimal control of infectious diseases via prevention and treatment” (Tech. Rep., Centre for Economic Policy Research (CEPR), 2012).

[19] R. Rowthorn, J. Maciejowski, A cost-benefit analysis of the COVID-19 disease. Oxf. Rev. Econ. Policy **36**, S38–S55 (2020). 10.1093/oxrep/graa030 PMC7499782 40504226

[20] M. Gersovitz, J. S. Hammer, The economical control of infectious diseases. Econ. J. **114**, 1–27 (2004).

[21] A. Z. Palmer, Z. B. Zabinsky, S. Liu, Optimal control of COVID-19 infection rate considering social costs. arXiv [Preprint] (2021). https://arxiv.org/abs/2007.13811 (Accessed 8 September 2025).

[22] J. Maciejowski, R. Rowthorn, S. Sheffield, D. Vines, A. Williamson, Mitigation policy for the covid-19 pandemic: Intertemporal optimisationusing an seir model. Available at SSRN 4003885 (2022). 10.2139/ssrn.4003885.

[23] S. Goldman, J. Lightwood, Cost optimization in the SIS model of infectious disease with treatment. Top. Econ. Anal. Policy **2**, 1007 (2002).

[24] F. Sélley, A. Besenyei, I. Z. Kiss, P. L. Simon, Dynamic control of modern, network-based epidemic models. SIAM J. Appl. Dyn. Syst. **14**, 168–187 (2015).

[25] T. Péni, B. Csutak, G. Szederkényi, G. Röst, Nonlinear model predictive control with logic constraints for COVID-19 management. Nonlinear Dyn. **102**, 1965–1986 (2020). 33281298 10.1007/s11071-020-05980-1 PMC7709478

[26] D. Martins, A. Bhaya, F. Pazos, N-step-ahead optimal control of a compartmental model of COVID-19. J. Control. Autom. Electr. Syst. **34**, 455–469 (2023).

[27] M. P. Lynch, S. K. Schnyder, J. J. Molina, R. Yamamoto, M. S. Turner, The theory of epidemics with altruism. Proc. Natl. Acad. Sci. U.S.A. **123**, e2518893123 (2026). 41758666 10.1073/pnas.2518893123 PMC12956880

[28] L. Bremaud, O. Giraud, D. Ullmo, Mean-field-game approach to nonpharmaceutical interventions in a social-structure model of epidemics. Phys. Rev. E **110**, 064301 (2024). 39916116 10.1103/PhysRevE.110.064301

[29] A. Q. Ohi, M. F. Mridha, M. M. Monowar, M. A. Hamid, Exploring optimal control of epidemic spread using reinforcement learning. Sci. Rep. **10**, 22106 (2020). 33328551 10.1038/s41598-020-79147-8 PMC7744528

[30] M. Arango, L. Pelov, COVID-19 pandemic cyclic lockdown optimization using reinforcement learning. arXiv [Preprint] (2020). 10.48550/arXiv.2009.04647 (Accessed 8 September 2025).

[31] Yg. Hwang, H. D. Kwon, J. Lee, Optimal control problem of various epidemic models with uncertainty based on deep reinforcement learning. Numer. Methods Partial. Differ. Equations **38**, 2142–2162 (2022).

[32] P. J. K. Libin *et al*., “Deep reinforcement learning for large-scale epidemic control” in *Machine Learning and Knowledge Discovery in Databases. Applied Data Science and Demo Track*, Y. Dong, G. Ifrim, D. Mladenić, C. Saunders, S. Van Hoecke, Eds. (Springer International Publishing, Cham, Switzerland, 2021), pp. 155–170.

[33] T. A. Perkins, G. España, Optimal control of the COVID-19 pandemic with non-pharmaceutical interventions. Bull. Math. Biol. **82**, 118 (2020). 32888118 10.1007/s11538-020-00795-y PMC7473596

[34] S. Contreras , The challenges of containing SARS-CoV-2 via test-trace-and-isolate. Nat. Commun. **12**, 378 (2021). 33452267 10.1038/s41467-020-20699-8 PMC7810722

[35] M. E. Kretzschmar, G. Rozhnova, M. Van Boven, Isolation and contact tracing can tip the scale to containment of COVID-19 in populations with social distancing. Front. Phys. **8**, 622485 (2021).

[36] S. A. Nowak, P. Nascimento, R. de Lima, Vardavas, Optimal non-pharmaceutical pandemic response strategies depend critically on time horizons and costs. Sci. Rep. **13**, 2416 (2023). 36765151 10.1038/s41598-023-28936-y PMC9912209

[37] S. Bauer , Relaxing restrictions at the pace of vaccination increases freedom and guards against further COVID-19 waves. PLoS Comput. Biol. **17**, 1–37 (2021). 10.1371/journal.pcbi.1009288 PMC8412259 34473693

[38] J. Bradbury , JAX: Composable transformations of Python+NumPy programs. *GitHub* (2018). https://github.com/jax-ml/jax. Accessed 8 September 2025.

[39] P. Kidger, “On Neural Differential Equations,” PhD thesis, University of Oxford, Oxford, UK (2021).

[40] R. M. Anderson, R. M. May, Infectious Diseases of Humans: Dynamics and Control (Oxford University Press, 1991).

[41] D. C. Liu, J. Nocedal, On the limited memory BFGS method for large scale optimization. Math. Prog. **45**, 503–528 (1989).

[42] J. Dehning, ICoMo (Inference of Compartmental Models). GitHub. https://github.com/Priesemann-Group/icomo. Accessed 23 April 2025.

[43] M. Moriyama, W. J. Hugentobler, A. Iwasaki, Seasonality of respiratory viral infections. Annu. Rev. Virol. **7**, 83–101 (2020). 32196426 10.1146/annurev-virology-012420-022445

[44] V. J. Hall , COVID-19 vaccine coverage in health-care workers in England and effectiveness of BNT162b2 mRNA vaccine against infection (SIREN): A prospective, multicentre, cohort study. Lancet **397**, 1725–1735 (2021). 33901423 10.1016/S0140-6736(21)00790-X PMC8064668

[45] M. Levine-Tiefenbrun , Initial report of decreased SARS-CoV-2 viral load after inoculation with the BNT162b2 vaccine. Nat. Med. **27**, 790–792 (2021). 33782619 10.1038/s41591-021-01316-7

[46] J. K. Peter , SARS-CoV-2 vaccine alpha and delta variant breakthrough infections are rare and mild but can happen relatively early after vaccination. Microorganisms **10**, 857 (2022). 35630302 10.3390/microorganisms10050857 PMC9146960

[47] T. Kompas, R. Q. Grafton, T. N. Che, L. Chu, J. Camac, Health and economic costs of early and delayed suppression and the unmitigated spread of COVID-19: The case of Australia. PLoS ONE **16**, e0252400 (2021). 34086731 10.1371/journal.pone.0252400 PMC8177447

[48] A. Pak , Economic consequences of the COVID-19 outbreak: The need for epidemic preparedness. Front. Public Health **8**, 241 (2020). 32574307 10.3389/fpubh.2020.00241 PMC7273352

[49] P. L. Sacco, F. Valle, M. De Domenico, Proactive vs. reactive country responses to the COVID-19 pandemic shock. PLoS Glob. Public Health **3**, e0001345 (2023). 36962977 10.1371/journal.pgph.0001345 PMC10021818

[50] M. Skovdal, M. Pickles, T. Hallett, C. Nyamukapa, S. Gregson, Complexities to consider when communicating risk of COVID-19. Public Health **186**, 283 (2020). 32871450 10.1016/j.puhe.2020.07.015 PMC7377722

[51] P. A. Maguire, J. C. Looi, Risk perception research informing recommendations for COVID-19 preventative health measures and public messaging. Australas. Psychiatry **30**, 601–603 (2022). 35938882 10.1177/10398562221117060 PMC9361031

[52] S. T. Heydari , The effect of risk communication on preventive and protective behaviours during the COVID-19 outbreak: Mediating role of risk perception. BMC Public Health **21**, 1–11 (2021). 33407302 10.1186/s12889-020-10125-5 PMC7787415

[53] F. N. Rahman , Challenges in preventive practices and risk communication towards COVID-19: A cross-sectional study in Bangladesh. Int. J. Environ. Res. Public Health **18**, 9259 (2021). 34501847 10.3390/ijerph18179259 PMC8430532

[54] J. Viana , Controlling the pandemic during the SARS-CoV-2 vaccination rollout. Nat. Commun. **12**, 3674 (2021). 34135335 10.1038/s41467-021-23938-8 PMC8209021

[55] B. Balmford, J. D. Annan, J. C. Hargreaves, M. Altoè, I. J. Bateman, Cross-country comparisons of covid-19: Policy, politics and the price of life. Environ. Resour. Econ. **76**, 525–551 (2020). 10.1007/s10640-020-00466-5 PMC7400753 32836862

[56] K. Sherratt , Predictive performance of multi-model ensemble forecasts of COVID-19 across European nations. eLife **12**, e81916 (2023). 37083521 10.7554/eLife.81916 PMC10238088

[57] R. E. Kalman, Contributions to the theory of optimal control. *Bol. Soc. Mat. Mexicana* **5**, 102–119 (1960).

[58] V. Priesemann , Calling for pan-European commitment for rapid and sustained reduction in SARS-CoV-2 infections. Lancet **397**, 92–93 (2021). 33347811 10.1016/S0140-6736(20)32625-8 PMC7833270

[59] L. Müller, Code for optimizing infectious disease mitigation under dynamic conditions. GitHub. https://github.com/Priesemann-Group/optimizing_pandemic_mitigation. Deposited 23 April 2025. 10.1073/pnas.2527395123 PMC13462870 42546195

