# Supplementary material for: Optimizing infectious disease mitigation under dynamic conditions
Source: Proc Natl Acad Sci U S A. 2026 Aug 3;123(32):e2527395123. doi: 10.1073/pnas.2527395123 (PMC13462870; doi:10.1073/pnas.2527395123)
Supplement: Supplementary file 1 [file pnas.2527395123.sapp.pdf]

Supporting Information for

Optimizing infectious disease mitigation under dynamic conditions

Laura Müller, Fabio Sartori, Jonas Dehning, Maximilian F. Eggl and Viola Priesemann

Viola Priesemann.
E-mail: viola.priesemannds.mpg.de

This PDF file includes:

- Supporting text
- Figs. S1 to S14
- Table S1
- SI References

Supporting Information Text

A. Extended version of theory and methods.

A.1. Implementation of the different scenarios. For the implementation of the disease dynamics, we use an SIRS model, which is described by Eq. (1), where β is the effective transmission rate, γ the recovery rate, and ν the waning rate. The mitigation M is included in the effective transmission rate: $\beta(t) = \beta_0(t)(1 - M(t))$.

$$\begin{aligned} \dot{S} &= -\beta(t)IS + \nu(1 - S - I) \\ \dot{I} &= +\beta(t)IS - \gamma I \end{aligned} \qquad [1]$$

The basic and effective reproduction numbers can be calculated as $R_0(t) = \beta_0(t)/\gamma$ and $R_{\mathsf{eff}}(t) = R_0(t)(1 - M(t))$, respectively.

The different scenarios are simulated by modifying the basic SIRS model. For the implementation of **seasonality**, we use a sinusoidal transmission rate (Eq. (2)), which oscillates around the mean value $\beta_{0,\mathsf{mean}}$ with an amplitude of $\Delta\beta$. The period is set to one year, so that the maximum and minimum of the sine function correspond to winter and summer, respectively.

$$\beta_0(t) = \beta_{0,\mathsf{mean}} + \Delta\beta \cdot \sin\left(2\pi \frac{t}{365}\right) \qquad [2]$$

To simulate **vaccination**, we split all three compartments into two compartments, each: one for *vaccinated* individuals, denoted by vac, and one for *unvaccinated* individuals (Eq. (4)-Eq. (9)). At a rate of N_{vac} (Eq. (3)) people are moved from the *unvaccinated* to the *vaccinated susceptible* compartment. This simulates a vaccination campaign. For the vaccination rate we chose a sigmoidal function as in the beginning only few vaccines are available and then an increasing number of people is able to get vaccinated each day. The plateau corresponds to ongoing booster vaccinations. As vaccinated people are less likely to get infected or infect others, we introduce the vaccine effectiveness η, which decreases the transmission rate if vaccinated people are involved. We assume waning immunity after vaccination, which happens at the rate ν_{vac}.

$$N_{\mathsf{vac}} = \frac{0.004}{1 + \exp(-0.1(t - 150))} \qquad [3]$$

$$\begin{aligned} dS =& -\beta(t)IS - \beta(t)(1-\eta)I_{\mathsf{vac}}S + \nu R \\ &+ \nu_{\mathsf{vac}}S_{\mathsf{vac}} - N_{\mathsf{vac}} && [4] \\ dS_{\mathsf{vac}} =& -\beta(t)(1-\eta)IS_{\mathsf{vac}} - \beta(t)(1-\eta)^2 I_{\mathsf{vac}}S_{\mathsf{vac}} \\ &+ \nu R_{\mathsf{vac}} - \nu_{\mathsf{vac}}S_{\mathsf{vac}} + N_{\mathsf{vac}} && [5] \\ dI =& \beta(t)IS + \beta(t)(1-\eta)I_{\mathsf{vac}}S - \gamma I && [6] \\ dI_{\mathsf{vac}} =& \beta(t)(1-\eta)IS_{\mathsf{vac}} + \beta(t)(1-\eta)^2 I_{\mathsf{vac}}S_{\mathsf{vac}} \\ &- \gamma I_{\mathsf{vac}} && [7] \\ dR =& \gamma I - \nu R && [8] \\ dR_{\mathsf{vac}} =& \gamma I_{\mathsf{vac}} - \nu R_{\mathsf{vac}} && [9] \end{aligned}$$

Apart from the different scenarios, the effective transmission rate is influenced by the mitigation (Eq. (4) in the main text). Mitigation includes both personal and government actions to slow the spread of infection. This strength can be realized by different combinations of measures. It can have value from 0 to 1, where 0 corresponds to no mitigation and 1 to full mitigation, meaning a complete stop of transmission.

As the time evolution of the optimal mitigation is not known, we implement it by setting one parameter for each time step ($\hat{=}$ 1 day), each of which can have a value between 0 and 1 and is independent of its neighbors. Therefore, the number of parameters needed to model the mitigation equals the simulation length in days, leading to a high-dimensional optimization problem.

A.2. Cost function. To find the parameters for the optimal mitigation, we need to define a cost function that we want to minimize. This cost function depends on the prevalence as well as the mitigation. The cost function is not expressed in real money values, but is just used for balancing the mitigation and infection costs. The optimal mitigation is defined as the mitigation strategy with the lowest total cost.

For the mitigation cost, we use a function that grows rapidly with increasing mitigation (Eq. (10), Fig. S1). It diverges, so that a mitigation value of 1 can never be reached, as this is unrealistic in real life. This cost includes social costs, like the mental effects of lockdowns, as well as economic costs, like the actual costs for masks, people not being able to work, etc.

$$C_M(t) = \frac{M}{1 - M} \qquad [10]$$

The shape of the infection cost can be split into two parts. It first grows quadratically until the prevalence reaches a value of $I \approx 0.001$, which can be interpreted as a hospitalization limit. Above this threshold, the cost grows linearly with a slope of five, as the healthcare system cannot provide the necessary treatments to everyone anymore. As we need a smooth transition between the two parts for the function to be differentiable, we use Eq. (11) (Fig. S1). The infection costs, as the mitigation costs, are comprised of social and economic costs. They include hospital costs but also costs for absence from work or even deaths.

$$C_I(t) = \frac{I}{v} + v\left(I(t) - 0.001\right) \cdot \left(1 - \frac{1}{1 + \exp(1000(I(t) - 0.001))}\right) + w, \quad [11]$$

where $v = 4.7913$, and $w = 0.0012885$ (v is chosen to obtain a smooth transition to a slope of 5 and w to ensure that $C_I(0) = 0$).

The total cost consists of the mitigation and infection cost added up and integrated over the whole simulation (Eq. (12)). The infection cost is multiplied by the disease severity, a, which defines the ratio between infection and mitigation cost. For harmless diseases, a is small, which means that the mitigation cost gets a higher relative weight, as we can tolerate high numbers of infectious people and do not need to invest in a lot of mitigation. For more severe diseases, on the other hand, a is high and we get a low weight for the mitigation cost, as we need a lot of mitigation because we do not want to risk lots of infections.

$$C_{\text{tot}} = \int_0^T \left(aC_I(I(t)) + C_M(M(t))\right) dt \quad [12]$$

It is important to note that the choice of the cost function and disease severity may depend on the disease as well as the personal assessment of a person, as everyone weighs the societal impact of the mitigation and seriousness of the prevalence differently.

Furthermore, to explore the robustness of our results, we employed a broader range of cost functions.

We tested the robustness of our seasonality, vaccination, and cost of waiting results in Supplementary Fig. S7, S11 and S13, respectively using two additional different mitigation costs, see Fig. S1-b.

$$C_{M,\text{log}} = -\log(1 - M), \quad [13]$$

$$C_{M,\text{sqrt}} = 2\left(\frac{1}{\sqrt{1 - M}} - 1\right). \quad [14]$$

We tested in Fig. S3 the dependence of the optimal mitigation on the hospitalization threshold using a sharp bi-linear simplification of the infection cost,

$$C_{I,\text{sharp}}(I) = \begin{cases} I & I \leq I_h, \\ I_h + 5\,(I - I_h) & I > I_h, \end{cases} \quad [15]$$

where I_h is the hospitalization threshold. This bi-linear form preserves the qualitative structure of the smooth cost in Eq. (11) – a mild slope below I_h and a steeper slope above – while removing the curvature of the smooth crossover, which makes the role of the threshold tractable analytically (see Fig. S1-c).

Finally in Fig. S6 we tested the robustness of the all-or-nothing transition across nine combinations of cost of infections and cost of mitigation:

$$C_I = I^{0.5}, \quad [16]$$

$$C_I = \text{as in Eq. 11}, \quad [17]$$

$$C_I = I^2, \quad [18]$$

see Fig. S1-d; and

$$C_M = \frac{M}{1 - M^{\zeta}}, \quad [19]$$

where $\zeta = 0.1, 1, 10$, see Fig. S1-e.

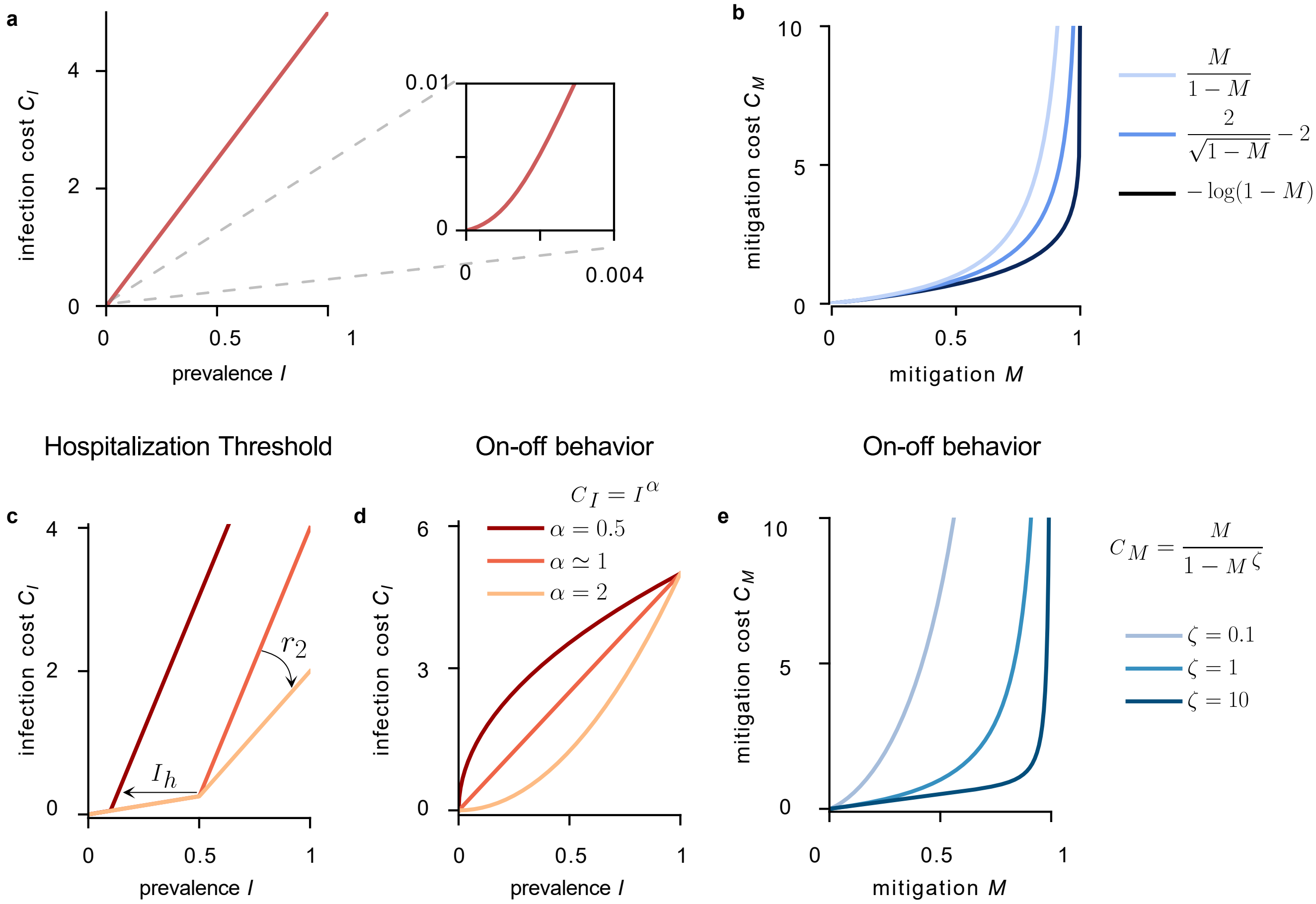

Fig. S1. Infection and mitigation costs. (a) The infection cost C_I used in the main paper as a function of prevalence I, showing approximately quadratic growth for small prevalence (inset) before transitioning to linear growth. **(b)** The three mitigation cost functions C_M used in the main paper (Eq. (10), Eq. (13), Eq. (14)). All functions diverge as $M \to 1$, reflecting the practical impossibility of complete transmission prevention. The divergence represents that while initial measures (hygiene, masks) are low-cost, achieving higher mitigation levels requires progressively more expensive interventions. The different growth rates illustrate the trade,offs between affordability and achievable mitigation levels: less steeply growing functions (darker) allow for higher levels of mitigation, while steeper ones (lighter) make extreme mitigation prohibitively costly, potentially making infections the cheaper alternative. **(c)** Sharp bilinear infection cost used to model a hospitalization threshold, with hospital capacity I_h marking the kink and slope r_2 controlling the cost above capacity. **(d)** Power-law infection costs $C_I = I^\alpha$ for $\alpha = 0.5, 2$, used in the analysis of all,or,nothing behavior. The case $\alpha \simeq 1$ corresponds to the (central, approximately linear) cost used in the main paper. **(e)** Generalized mitigation cost $C_M = M/(1 - M^\zeta)$ for $\zeta = 0.1, 1, 10$, used to probe how the steepness of the divergence near $M = 1$ affects the all,or,nothing structure.

A.3. Parameters. All parameter information for the SIRS model and scenarios can be found in Tab. S1.

A.4. End of simulation. To ensure consistent comparison across scenarios, we implement a controlled conclusion to each simulation (i.e. Fig. S4). We introduce an exponential decrease of the basic reproduction number to 0 over the final 90 days, which can be interpreted as the natural endpoint of epidemic dynamics due to accumulated population immunity.

This approach serves multiple purposes: First, it allows the epidemic to reach a natural conclusion within the simulation time frame rather than artificially truncating ongoing dynamics. Second, it ensures that mitigation strategies across different scenarios converge in a comparable manner, facilitating meaningful comparisons between results.

B. Mitigation threshold. At any point in time, the infections can either grow, decay or stay constant. Assuming the parameters β, γ, and ν are constant, the change over time in $\frac{dI(t)}{dt}$ depends on the susceptible fraction $S(t)$ and mitigation $M(t)$. From this, we can calculate the mitigation stringency M_{thresh} that defines the growth-decay boundary for a given $S(t)$. For $M > M_{\text{thresh}}$, infections decay, for $M < M_{\text{thresh}}$, they increase.

In particular, starting from the infected compartment in the SIRS system (Eq. (4) in the main text), the growth-decay boundary is defined by $\frac{dI}{dt} = 0$ for $I(t) > 0$, which yields

$$0 = \beta(1 - M_{\text{thresh}}(t))S(t) - \gamma. \quad [20]$$

Solving for $M_{\text{thresh}}(t)$ gives

$$M_{\text{thresh}}(t) = 1 - \frac{\gamma}{\beta S(t)}. \quad [21]$$

Table S1. Default values for parameters. Results for other values can be found in Supplementary Sec. H. As we do not simulate a specific disease in this paper, the values are assumed. The recovery rate is chosen to be similar to COVID-19 (1), and the waning rate of immunity after infection is a little faster than for COVID-19 (2).

Parameter	Symbol	Unit	Default value
disease severity	a	-	10
basic reproduction number	R_0	-	2
seasonal fluctations	ΔR_0	-	1
recovery rate	γ	day^{-1}	0.1
immunity waning rate after infection	ν	day^{-1}	0.01
immunity waning rate after vaccination	ν_{vac}	day^{-1}	1/150
vaccine effectiveness	η	-	$\in [0, 1]$
mitigation delay	δt	days	$\in [0, 200]$
hospital capacity	I_h	-	0.001
pre-hospital capacity slope	r_1	-	1
post-hospital capacity slope	r_2	-	5
cost of infection exponent	α	-	$\in \{0.5, 2\}$
cost of mitigation exponent	ζ	-	$\in \{0.1, 1, 10\}$

Using $R_0(t) = \beta(t)/\gamma$, this can be written as

$$M_{\text{thresh}}(t) = 1 - \frac{1}{R_0(t)\,S(t)}. \quad [22]$$

C. Analytical calculation of critical disease severity and optimal mitigation stringency. Our results show that in the absence of any specific scenarios like seasonality, the optimal mitigation is constant (see Fig. 1 in the main textd-f). Under this assumption ($M(t) = M$), we can calculate the optimal mitigation stringency $M_{\text{opt}}(a)$ as a function of the model parameters and disease severity, as well as the critical disease severity a_{crit}, at which the abrupt transition in the optimal stringency occurs.

For this, we first calculate the fixed points of our model (Eq. (4) in the main text), denoted by *, by setting the derivatives of the compartments to zero:

$$\begin{aligned}
0 &= -R_0(1-M)I^*S^* + \frac{\nu}{\gamma}R^*,\\
0 &= R_0(1-M)I^*S^* - I^*,\\
0 &= I^* - \frac{\nu}{\gamma}R^*,
\end{aligned}$$

where $R_0 = \beta_0/\gamma$. The trivial solution is the disease-free equilibrium $S^* = 1$, $I^* = 0$, $R^* = 0$. For the endemic equilibrium with $I^* \neq 0$, using $S^* + I^* + R^* = 1$, we obtain

$$\begin{aligned}
S^* &= \frac{1}{R_0(1-M)},\\
I^* &= \frac{\nu\left(1 - \frac{1}{R_0(1-M)}\right)}{\gamma + \nu},\\
R^* &= \frac{\gamma}{\nu}I^*.
\end{aligned} \quad [23]$$

For the endemic equilibrium to be physically meaningful, we require $I^* > 0$. From Eq. (23), this implies

$$1 - \frac{1}{R_0(1-M)} > 0,$$

which yields the condition

$$R_0(1-M) > 1.$$

Hence, the endemic equilibrium exists only if the effective reproduction number $R_{\text{eff}} = R_0(1-M)$ exceeds unity. Otherwise, the only feasible equilibrium is the disease-free state.

From this condition, we can define a threshold mitigation level $\tilde{M}$ separating the two regimes by solving $R_0(1-M) = 1$, which gives

$$\tilde{M} = 1 - \frac{1}{R_0}.$$

This is equivalent to the growth-decay boundary with $S = 1$ (see Sec. B). For $M < \tilde{M}$, the system admits an endemic equilibrium with $I^* > 0$, whereas for $M \geq \tilde{M}$, the disease-free equilibrium is the only physically meaningful solution.

Now that we have the infections as a function of the mitigation, $I^*(M)$, we can write the infection cost, and therefore the total cost, as a function of mitigation strength.

$$C_{\text{tot}}(M) = \int_0^T (aC_I(M) + C_M(M))dt$$

As we are in the equilibrium, the integral reduces to a constant factor, τ, which gives us

$$C_{\text{tot}}(M) = \tau C(M).$$

Because our cost is defined in arbitrary units, we can omit τ and concentrate on $C(M)$. Assuming a linear infection cost function $C_I = I$, we obtain

$$C(M) = \begin{cases} a\frac{\nu\left(1 - \frac{1}{R_0(1-M)}\right)}{\gamma+\nu} + \frac{M}{1-M} & M \leq \tilde{M},\\ \frac{M}{1-M} & M \geq \tilde{M}. \end{cases}$$

The optimal mitigation M_{opt} is defined as the mitigation with the least total cost. Because $C(M)$ is not differentiable everywhere, however, we cannot simply set its derivative to 0 to obtain the minimum.

Let us start with the piece-wise derivative:

$$\frac{dC(M)}{dM} = \begin{cases} \frac{1}{(1-M)^2}\left(1 - \frac{a\nu}{(\gamma+\nu)R_0}\right) & M \leq \tilde{M}, \\ \frac{1}{(1-M)^2} & M \geq \tilde{M}. \end{cases}$$

We can see that for $M \geq \tilde{M}$, the derivative is always positive, meaning the minimum depends on the slope for $M \leq \tilde{M}$, which is dependent on a:

$$\begin{aligned} a < \frac{(\gamma+\nu)R_0}{\nu} \quad &\rightarrow \quad \frac{dC(M)}{dM} > 0, \\ a = \frac{(\gamma+\nu)R_0}{\nu} \quad &\rightarrow \quad \frac{dC(M)}{dM} = 0, \\ a > \frac{(\gamma+\nu)R_0}{\nu} \quad &\rightarrow \quad \frac{dC(M)}{dM} < 0. \end{aligned}$$

For $a < \frac{(\gamma+\nu)R_0}{\nu}$, the slope of $C(M)$ is positive over the whole range of M, meaning the optimal mitigation is a boundary solution at $M_{\text{opt}} = 0$. For $a > \frac{(\gamma+\nu)R_0}{\nu}$, on the other hand, the slope is negative for $M < \tilde{M}$ and positive for $M > \tilde{M}$, meaning $M_{\text{opt}} = \tilde{M}$. As $a = \frac{(\gamma+\nu)R_0}{\nu}$ is the point at which the minimum switches, we define it as the critical disease severity

$$a_{\text{crit}} = \frac{(\gamma+\nu)R_0}{\nu}.$$

For all disease severities higher than a_{crit}, we have the same optimal mitigation stringency, while, for all disease severities smaller than a_{crit}, the optimal choice is not to mitigate at all. In short:

$$\begin{aligned} a < a_{\text{crit}} \quad &\rightarrow \quad M_{\text{opt}} = 0, \\ a > a_{\text{crit}} \quad &\rightarrow \quad M_{\text{opt}} = 1 - \frac{1}{R_0}. \end{aligned}$$

In the case of time-dependent mitigation, the optimal mitigation for $a > a_{\text{crit}}$ follows the growth-decay boundary M_{thresh}, as shown in Fig. S2.

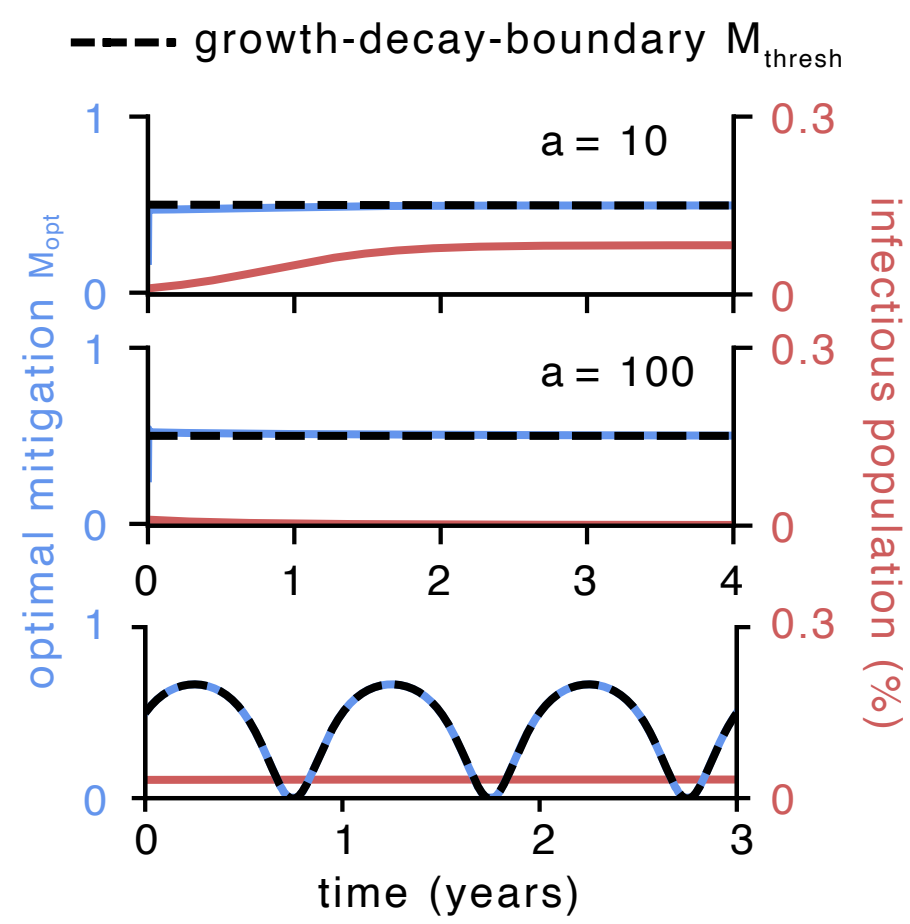

Fig. S2. The optimal mitigation (blue) follows the growth-decay boundary (black dashed). The first two plots show the general case without any scenarios for two different disease severities a. The third plot shows the optimal mitigation in the presence of seasonality in the case with the logarithmic mitigation cost function $(\text{Eq. } (13))$. This choice is because, in contrast to the standard mitigation cost function (Eq. (2) in the main text, the optimal mitigation with the logarithmic cost is able to completely counteract the seasonality, leading to constant infections.

D. Generalization to a broader class of mitigation costs. The proof in Sec. C relies on the specific form $C_M(M) = M/(1-M)$. We now show that the all-or-nothing structure persists for a broader class of mitigation costs, provided the infection cost is linear in prevalence ($C_I = I$) and C_M satisfies a mild growth condition that we make precise below.

As before, we restrict to constant mitigation $M(t) = M$ and work at the endemic equilibrium. From Eq. (23), the equilibrium prevalence as a function of M is

$$I^*(M) = \begin{cases} \dfrac{\nu\left(1 - \dfrac{1}{R_0(1-M)}\right)}{\gamma+\nu} & M \le \tilde{M}, \\ 0 & M \ge \tilde{M}, \end{cases}$$

with $\tilde{M} = 1 - 1/R_0$.

Dropping the equilibrium time factor τ, the total cost as a function of M becomes

$$C(M) = \begin{cases} a\,\dfrac{\nu\left(1 - \dfrac{1}{R_0(1-M)}\right)}{\gamma+\nu} + C_M(M) & M \le \tilde{M}, \\ C_M(M) & M \ge \tilde{M}. \end{cases}$$

We assume C_M is strictly increasing and differentiable on $[0,1)$. The piecewise derivative reads

$$\frac{dC(M)}{dM} = \begin{cases} -\dfrac{a\,\nu}{(\gamma+\nu)R_0(1-M)^2} + C_M'(M) & M \le \tilde{M}, \\ C_M'(M) & M \ge \tilde{M}. \end{cases}$$

Step 1: ruling out the regime $M > \tilde{M}$. For $M \ge \tilde{M}$, $C_M'(M) > 0$, so the derivative is strictly positive: increasing mitigation beyond $\tilde{M}$ only adds cost without further reducing infections, since $I^* = 0$ already. Hence the optimum cannot lie in $(\tilde{M}, 1)$, and the candidates are restricted to $[0, \tilde{M}]$.

Step 2: ruling out an interior minimum on $(0, \tilde{M})$. For $M \le \tilde{M}$, the derivative contains a negative term, $-a\nu/[(\gamma+\nu)R_0(1-M)^2]$, and a positive term, $C_M'(M)$. An interior minimum at some $M^* \in (0, \tilde{M})$ would require dC/dM to change sign from negative to positive as M increases through M^*. We will impose a condition on C_M that prevents this.

Multiplying dC/dM by $(1-M)^2 > 0$ (which preserves its sign):

$$(1-M)^2 \frac{dC(M)}{dM} = -\frac{a\,\nu}{(\gamma+\nu)R_0} + g(M), \qquad g(M) := C_M'(M)(1-M)^2.$$

The first term is constant in M. Therefore, on $[0, \tilde{M}]$, the sign of dC/dM is determined by whether $g(M)$ exceeds the constant threshold $a\nu/[(\gamma+\nu)R_0]$.

We now impose the assumption that

$$g(M) = C_M'(M)(1-M)^2 \text{ is non-increasing on } [0, \tilde{M}]. \qquad [24]$$

Under this assumption, the function $M \mapsto (1-M)^2\, dC/dM = g(M) - a\nu/[(\gamma+\nu)R_0]$ is non-increasing in M. This implies that dC/dM itself, having the same sign as $g(M) - a\nu/[(\gamma+\nu)R_0]$, cannot transition from negative to positive as M increases. Three cases are possible:

- $g(M) > a\nu/[(\gamma+\nu)R_0]$ throughout $[0, \tilde{M}]$: then $dC/dM > 0$ everywhere, and C is strictly increasing. The optimum is $M_{\text{opt}} = 0$.

- $g(M) < a\nu/[(\gamma+\nu)R_0]$ throughout $[0, \tilde{M}]$: then $dC/dM < 0$ everywhere, and C is strictly decreasing. The optimum is $M_{\text{opt}} = \tilde{M}$.

- $g(M)$ crosses the threshold $a\nu/[(\gamma+\nu)R_0]$ once from above: then dC/dM goes from positive to negative, and C has an interior *maximum*, not a minimum. The optimum lies at one of the endpoints $\{0, \tilde{M}\}$.

In all three cases, the optimum lies at the boundary, $M_{\text{opt}} \in \{0, \tilde{M}\}$, and no interior optimum can exist.

Step 3: comparing the two boundary candidates. To decide between $M_{\text{opt}} = 0$ and $M_{\text{opt}} = \tilde{M}$, we compare $C(0)$ and $C(\tilde{M})$ directly. Using $I^*(0) = \nu(1 - 1/R_0)/(\gamma + \nu)$ and $I^*(\tilde{M}) = 0$, and choosing $C_M(0) = 0$ without loss of generality,

$$C(0) = a\,\frac{\nu\left(1 - \dfrac{1}{R_0}\right)}{\gamma + \nu},$$
$$C(\tilde{M}) = C_M(\tilde{M}).$$

The condition $C(\tilde{M}) < C(0)$ becomes

$$a > \frac{(\gamma + \nu)\,C_M(\tilde{M})}{\nu\left(1 - \dfrac{1}{R_0}\right)} = \frac{(\gamma + \nu)\,R_0\,C_M(\tilde{M})}{\nu(R_0 - 1)},$$

which defines the generalized critical disease severity

$$a_{\text{crit}} = \frac{(\gamma + \nu)\,R_0\,C_M(\tilde{M})}{\nu(R_0 - 1)}.$$

In summary,

$$a < a_{\text{crit}} \quad \rightarrow \quad M_{\text{opt}} = 0,$$
$$a > a_{\text{crit}} \quad \rightarrow \quad M_{\text{opt}} = \tilde{M} = 1 - \frac{1}{R_0}.$$

The all-or-nothing transition is therefore not specific to the form $C_M(M) = M/(1-M)$: it is a structural consequence of (i) the linear infection cost combined with the kink in $I^*(M)$ at $\tilde{M}$, and (ii) condition Eq. (24), which requires $C'_M(M)(1-M)^2$ to be non-increasing on $[0, \tilde{M}]$. This condition is satisfied by a broad class of mitigation costs, including the linear cost $C_M(M) = M$, the default cost $C_M(M) = M/(1 - M)$ (for which $g(M) = 1$), and more generally the family $C_M(M) = M/(1 - M^\zeta)$ for any $\zeta \geq 1$. Different choices of C_M shift the value of a_{crit} through the factor $C_M(\tilde{M})$, but do not alter the binary structure of the optimal strategy.

Consistency check. For the original case $C_M(M) = M/(1 - M)$, we have $C'_M(M) = 1/(1 - M)^2$, hence $g(M) = 1$, which is trivially non-increasing, so condition Eq. (24) holds. Moreover, $C_M(\tilde{M}) = (1 - 1/R_0)/(1/R_0) = R_0 - 1$, which yields

$$a_{\text{crit}} = \frac{(\gamma + \nu)\,R_0\,(R_0 - 1)}{\nu\,(R_0 - 1)} = \frac{(\gamma + \nu)R_0}{\nu},$$

recovering the result of Sec. C.

E. Generalization to power-law infection costs. We now extend the analysis of Sec. D to a broader class of infection costs of the form $C_I(I) = I^\alpha$ with $\alpha > 0$, while retaining the assumption that C_M satisfies condition Eq. (24). We show that the all-or-nothing structure persists for all $\alpha \leq 1$, while the method of this proof breaks down for $\alpha > 1$.

As before, we restrict to constant mitigation $M(t) = M$ and work at the endemic equilibrium. The total cost as a function of M becomes

$$C(M) = \begin{cases} a\,[I^*(M)]^\alpha + C_M(M) & M \leq \tilde{M}, \\ C_M(M) & M \geq \tilde{M}, \end{cases}$$

with $I^*(M)$ given by Eq. (23) and $\tilde{M} = 1 - 1/R_0$.

Step 1: ruling out the regime $M > \tilde{M}$. Identical to Sec. D: for $M \geq \tilde{M}$, $I^* = 0$ and $C(M) = C_M(M)$ is strictly increasing, so the optimum cannot lie in $(\tilde{M}, 1)$.

Step 2: ruling out an interior minimum on $(0, \tilde{M})$. Using $dI^*/dM = -\nu/[(\gamma + \nu)R_0(1 - M)^2]$, the derivative on $[0, \tilde{M}]$ reads

$$\frac{dC(M)}{dM} = -\frac{a\,\alpha\,\nu\,[I^*(M)]^{\alpha-1}}{(\gamma + \nu)R_0(1 - M)^2} + C'_M(M).$$

Multiplying by $(1 - M)^2 > 0$ to preserve sign:

$$(1 - M)^2\,\frac{dC(M)}{dM} = g(M) - \phi(M),$$

with $g(M) := C'_M(M)(1 - M)^2$ as before, and

$$\phi(M) := \frac{a\,\alpha\,\nu}{(\gamma + \nu)R_0}\,[I^*(M)]^{\alpha-1}.$$

The crucial difference with respect to Sec. D is that $\phi(M)$ is no longer constant in M: it inherits an M-dependence through $[I^*(M)]^{\alpha-1}$.

For an interior minimum to exist on $(0, \tilde{M})$, dC/dM must transition from negative to positive as M increases. This is ruled out if $g(M) - \phi(M)$ is non-increasing on $[0, \tilde{M}]$, because a non-increasing function can cross zero at most once and only from positive to negative.

By assumption Eq. (24), $g(M)$ is non-increasing. Hence a sufficient condition is that $\phi(M)$ is non-decreasing on $[0, \tilde{M}]$. Since $I^*(M)$ is strictly decreasing on $[0, \tilde{M}]$ (more mitigation reduces equilibrium prevalence), the monotonicity of ϕ depends on the sign of $\alpha - 1$:

- If $\alpha < 1$, then $[I^*(M)]^{\alpha-1}$ is increasing in M, so ϕ is non-decreasing. The proof goes through.
- If $\alpha = 1$, then $[I^*(M)]^{\alpha-1} \equiv 1$, ϕ is constant, and we recover Sec. D.
- If $\alpha > 1$, then $[I^*(M)]^{\alpha-1}$ is decreasing in M, so ϕ is decreasing. The function $g(M) - \phi(M)$ is then a difference of two non-increasing functions, whose monotonicity is not determined, and the argument breaks down.

We therefore conclude that for $\alpha \le 1$, no interior minimum can exist on $(0, \tilde{M})$, and the optimum lies at one of the boundary points $\{0, \tilde{M}\}$.

Step 3: comparing the two boundary candidates. For $\alpha \le 1$, the comparison proceeds as in Sec. D, with $[I^*(0)]^\alpha = [\nu(1 - 1/R_0)/(\gamma + \nu)]^\alpha$ replacing the linear term. Setting $C_M(0) = 0$,

$$C(0) = a \left[\frac{\nu \left(1 - \frac{1}{R_0}\right)}{\gamma + \nu} \right]^\alpha,$$

$$C(\tilde{M}) = C_M(\tilde{M}).$$

The condition $C(\tilde{M}) < C(0)$ defines the generalized critical disease severity

$$a_{\text{crit}} = C_M(\tilde{M}) \left[\frac{\gamma + \nu}{\nu \left(1 - \frac{1}{R_0}\right)} \right]^\alpha = \frac{C_M(\tilde{M})\, (\gamma + \nu)^\alpha\, R_0^\alpha}{\nu^\alpha\, (R_0 - 1)^\alpha},$$

which reduces to the result of Sec. D for $\alpha = 1$.

Summary. For any $\alpha \le 1$ and any C_M satisfying condition Eq. (24), the all-or-nothing transition persists:

$$a < a_{\text{crit}} \quad \rightarrow \quad M_{\text{opt}} = 0,$$
$$a > a_{\text{crit}} \quad \rightarrow \quad M_{\text{opt}} = \tilde{M} = 1 - \frac{1}{R_0}.$$

Remark on $\alpha > 1$. The sufficient condition derived above does not extend to $\alpha > 1$. This does not imply that the all-or-nothing structure necessarily fails for superlinear infection costs, but only that the present argument is insufficient to establish it. In this regime, $\phi(M)$ becomes a decreasing function of M, reflecting the fact that the marginal benefit of additional mitigation grows as prevalence approaches zero. This opens the possibility that the marginal infection cost remains too low at small M to justify mitigation, yet becomes large enough at intermediate M to make further mitigation worthwhile, allowing an interior minimum of $C(M)$ to emerge. This is consistent with the empirical results in Fig. S6, where superlinear infection costs ($p = 2$) combined with a milder mitigation cost produce a smooth dependence of M_{opt} on disease severity rather than an abrupt transition.

F. Joint dependence on infection and mitigation cost shapes. The analyses of Sec. D and Sec. E provide two independent sufficient conditions for the all-or-nothing behavior:

$$\text{(C1)} \quad g(M) = C'_M(M)(1 - M)^2 \text{ is non-increasing on } [0, \tilde{M}],$$
$$\text{(C2)} \quad \phi(M) = \tfrac{a\,\alpha\,\nu}{(\gamma+\nu)R_0} [I^*(M)]^{\alpha-1} \text{ is non-decreasing on } [0, \tilde{M}].$$

For $C_I = I^\alpha$ and $C_M = M/(1 - M^\zeta)$, (C1) holds iff $\zeta \ge 1$ and (C2) holds iff $\alpha \le 1$. When both hold, the all-or-nothing structure is guaranteed.

When one or both conditions fail, our proof no longer applies, but this does not in itself imply that the all-or-nothing structure breaks down: the conditions are sufficient, not necessary. In particular, $g(M) - \phi(M)$ may still happen to be non-increasing, or may cross zero only once from positive to negative, in which case the optimum still lies at the boundary. Establishing or ruling out the all-or-nothing behavior outside the regime covered by (C1) and (C2) therefore requires either a different argument or direct numerical inspection of $C(M)$.

Empirically, we found few cases where the all-or-nothing structure breaks down: for $\alpha = 2$ and $\zeta = 0.1, 1$ and for $alpha = 1$, and $\zeta = 0.1$ S6.

Consequences for the family $C_M = M/(1 - M^\zeta)$. For the mitigation cost family used in the main text and in S6, $C_M(M) = M/(1 - M^\zeta)$, condition (C1) reduces to a simple requirement on ζ. Computing

$$C_M'(M) = \frac{1 - M^\zeta + \zeta M^\zeta}{(1 - M^\zeta)^2} = \frac{1 + (\zeta - 1)M^\zeta}{(1 - M^\zeta)^2},$$

we obtain

$$g(M) = C_M'(M)(1 - M)^2 = \frac{[1 + (\zeta - 1)M^\zeta](1 - M)^2}{(1 - M^\zeta)^2}.$$

With a numerical inspection we can observe that the condition (C1) is satisfied for For $\zeta \geq 1$.

Combined with (C2), which holds for $\alpha \leq 1$ and fails for $\alpha > 1$, this means that for the family $C_M = M/(1 - M^\zeta)$ with $C_I = I^\alpha$, the all-or-nothing structure is guaranteed by our proof whenever $\zeta \geq 1$ and $\alpha \leq 1$. In particular, the standard choice of the main text, $\zeta = 1$ and $\alpha = 1$, sits inside this regime, and the linear infection cost case analyzed in Sec. C is recovered as the boundary of both conditions. Outside this regime, the proof is silent: the all-or-nothing structure may still hold or may break down, and, for example, S6-c with $\alpha = 2$, $\zeta = 0.1$ provides a clear example where the all-or-nothing regime break down.

G. Soft pinning at the hospitalization threshold. A natural question is whether the optimal mitigation strategy ever stabilizes the dynamics at or just below the hospitalization threshold I_h, where the marginal infection cost is lowest. This question is partially addressed by the theorems of Sec. D and Sec. E: under conditions (C1) and (C2), which require the infection cost to be concave (or linear) and the mitigation cost to be sufficiently steep, the equilibrium cost $C(M)$ admits no interior minimum, and the optimum lies at one of the boundary points $\{0, \tilde{M}\}$. The infection cost used in the main text, however, lies outside this regime, leaving open the possibility of an interior optimum at or near I_h.

To explore this possibility, we consider a sharp piecewise version of the cost, Eq. (15), with a bi-linear shape: slope 1 below I_h and slope 5 above. This bi-linear simplification preserves the qualitative structure of the smooth cost while making the role of the threshold tractable analytically. Because the equilibrium cost $C(M)$ is now piecewise built from $C_M(M) = M/(1 - M)$ plus a piecewise-linear function of $I^*(M)$, no curvature-driven interior minima can arise, and the only candidate optima are the kinks: $\{0, M_h, \tilde{M}\}$, where M_h pins the equilibrium prevalence exactly at I_h. Direct comparison of $C(0)$, $C(M_h)$, and $C(\tilde{M})$ identifies three regimes in the disease severity a: for low a, no mitigation is optimal; for intermediate a, pinning at I_h becomes the cheapest strategy; for high a, full suppression at $\tilde{M}$ takes over (Fig. S3a, b, dashed).

Replacing the bi-linear cost with the smooth form used in the main text Eq. (11) regularizes the transitions (Fig. S3a, b, solid): the discrete jump in C_I' at I_h is replaced by a smooth crossover, and the lock at I_h is replaced by a smooth decrease of I^*_{opt} from slightly above I_h to zero, with the subsequent transition to full suppression becoming gradual rather than abrupt. The all-or-nothing structure is therefore not violated, but rather softened in a controlled way that reflects the absence of a true kink in the infection cost.

The three-regime structure of the bi-linear cost is robust across hospitalization thresholds. Varying I_h across three orders of magnitude (Fig. S3c, d), the three regimes – no mitigation, pinning at I_h, full suppression – persist for all values of I_h explored, with only the mitigation level in the pinning regime depending on I_h.

The pinning mechanism becomes most explicit when we sweep I_h at fixed disease severity (Fig. S3e, f). At low a ($a = 1$), no mitigation is optimal regardless of I_h. At intermediate a ($a = 10$), the pinning regime is active: M_{opt} tracks the value $M_h(I_h)$ that locks prevalence at I_h, and I^*_{opt} rides the diagonal $I^*_{\text{opt}} = I_h$ until it saturates at I_{unmit}, beyond which pinning is no longer meaningful since the threshold lies above the unmitigated endemic prevalence. At high a ($a = 100$), the system is already in the full-suppression regime and the optimum is independent of I_h. This confirms that pinning is a genuine equilibrium-level phenomenon, but a narrow one: it requires the disease severity to lie in a specific intermediate window, with the hospitalization threshold below the unmitigated prevalence. Outside this window, the optimum collapses back to one of the all-or-nothing boundaries predicted by the theorems.

This three-regime structure is consistent with the behavior of the optimal mitigation in the main-text Fig. 1 in the main text-g, obtained with the smooth infection cost: the sharp transition out of $M_{\text{opt}} = 0$ marks the boundary between the no-mitigation and pinning regimes, and the inset reveals the regularized pinning shoulder of I^*_{opt} near I_h before its decrease to zero in the full-suppression regime.

H. Additional plots.

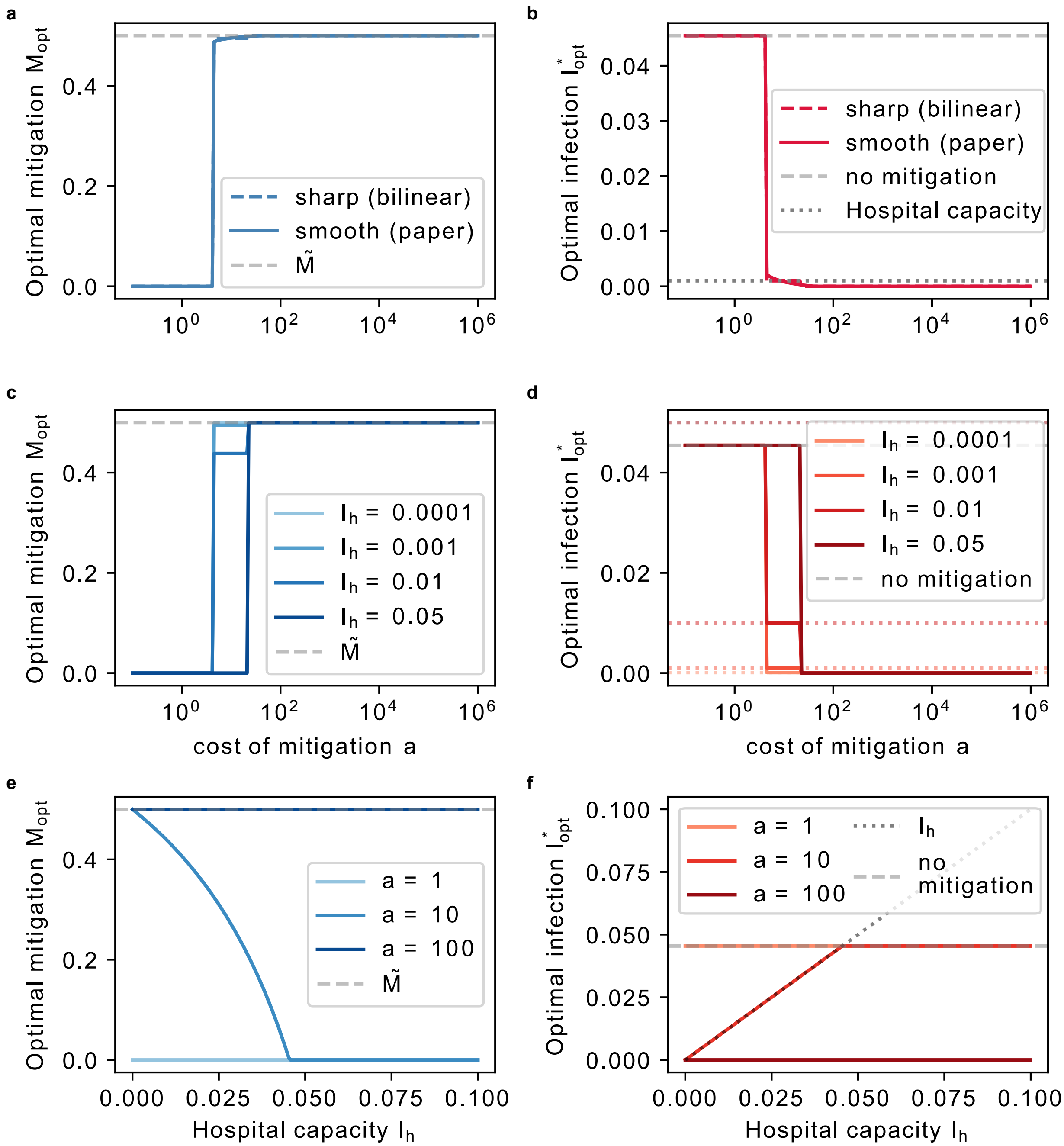

Fig. S3. Hospitalization threshold induces a three-regime structure in the optimal mitigation strategy. (a, b) Optimal mitigation M_{opt} and corresponding equilibrium prevalence I^*_{opt} as a function of disease severity a, for the bilinear sharp infection cost (dashed, see Eq. 15) and the smooth infection cost used in the main text (solid), both with the hospitalization threshold fixed at $I_h = 0.001$. The bilinear cost has slope 1 below I_h and slope 5 above; the smooth cost regularizes the kink at I_h. Both costs yield a sharp transition out of $M_{\text{opt}} = 0$ followed by a brief pinning regime at $I^*_{\text{opt}} = I_h$, before transitioning to full suppression $M_{\text{opt}} = \tilde{M}$. **(c, d)** Same as (a, b) for the bilinear sharp cost only, varying the hospitalization threshold $I_h \in \{10^{-4}, 10^{-3}, 10^{-2}, 5 \cdot 10^{-2}\}$. Three regimes are visible: for low a, no mitigation is optimal ($M_{\text{opt}} = 0$, $I^*_{\text{opt}} = I_{\text{unmit}}$); for intermediate a, the optimum pins prevalence at the threshold ($I^*_{\text{opt}} = I_h$, dotted lines); for high a, full suppression takes over ($M_{\text{opt}} = \tilde{M}$, $I^*_{\text{opt}} = 0$). **(e, f)** Sweep over I_h at three fixed values of disease severity $a \in \{1, 10, 100\}$, using the bilinear sharp cost 15. At $a = 1$, the system sits in the no-mitigation regime for all I_h in the range. At $a = 10$, the pinning regime is active: M_{opt} tracks the value $M_h(I_h)$ that locks prevalence at I_h, and I^*_{opt} rides the diagonal $I^*_{\text{opt}} = I_h$ (dotted) until it saturates at I_{unmit}. At $a = 100$, the system is already in the full-suppression regime and $M_{\text{opt}} = \tilde{M}$, $I^*_{\text{opt}} = 0$ independently of I_h. Parameters: $R_0 = 2$, $\gamma = 0.1$, $\nu = 0.01$.

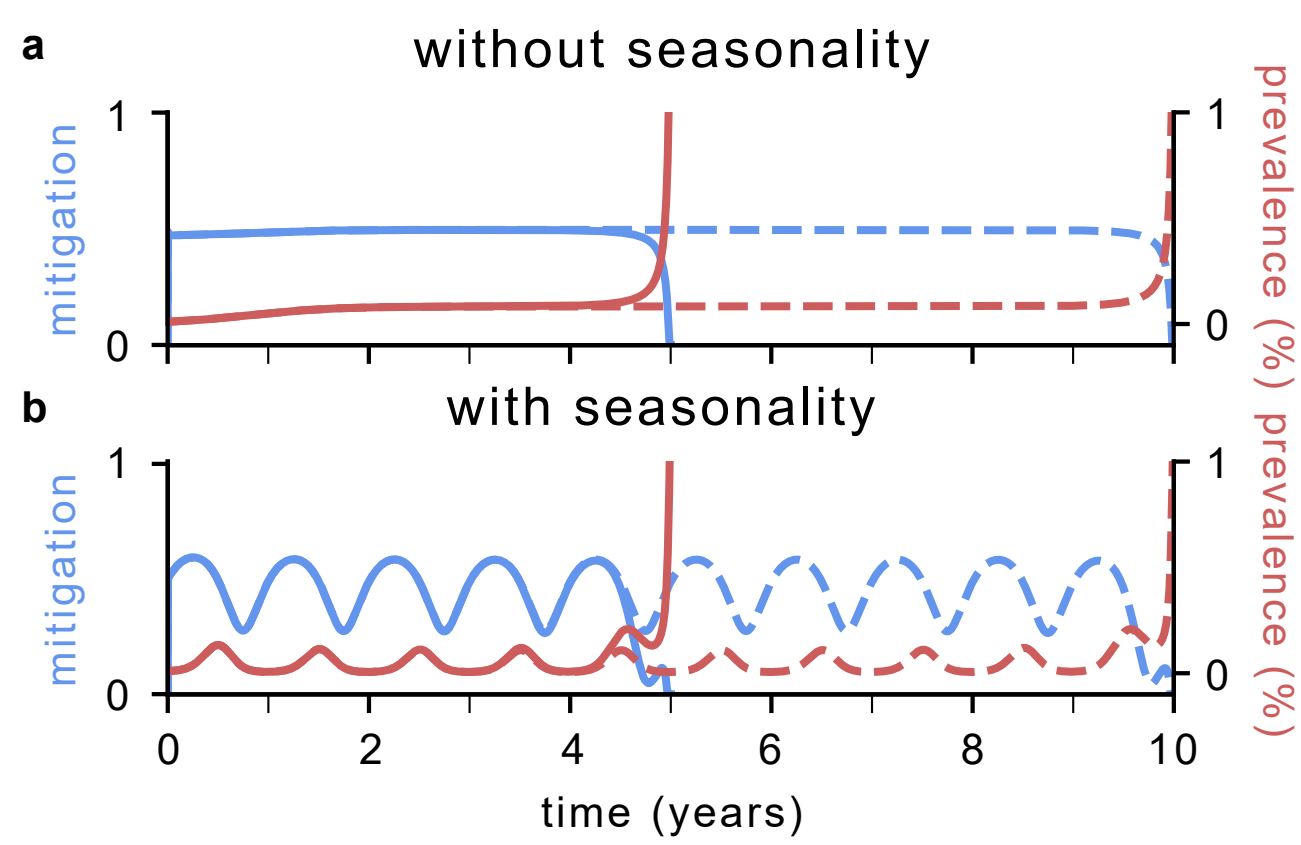

Fig. S4. Comparison between different simulation lengths. To show that our results are not artifacts due to the simulation length, we compared two scenarios that differ only in their duration one lasting 5 years (solid lines) and the other 10 years (dashed lines). The outcomes of both simulations are overlaid for comparison. Panel **a** displays mitigation and prevalence for a scenario where the reproduction number is constant, while panel **b** shows the mitigation and prevalence as a function of time in the seasonality scenario. In both cases, the results are very similar for the two simulation lengths. Only near the end of each simulation, boundary effects become preponderant, as discussed in Supplementary Sec. A.4.

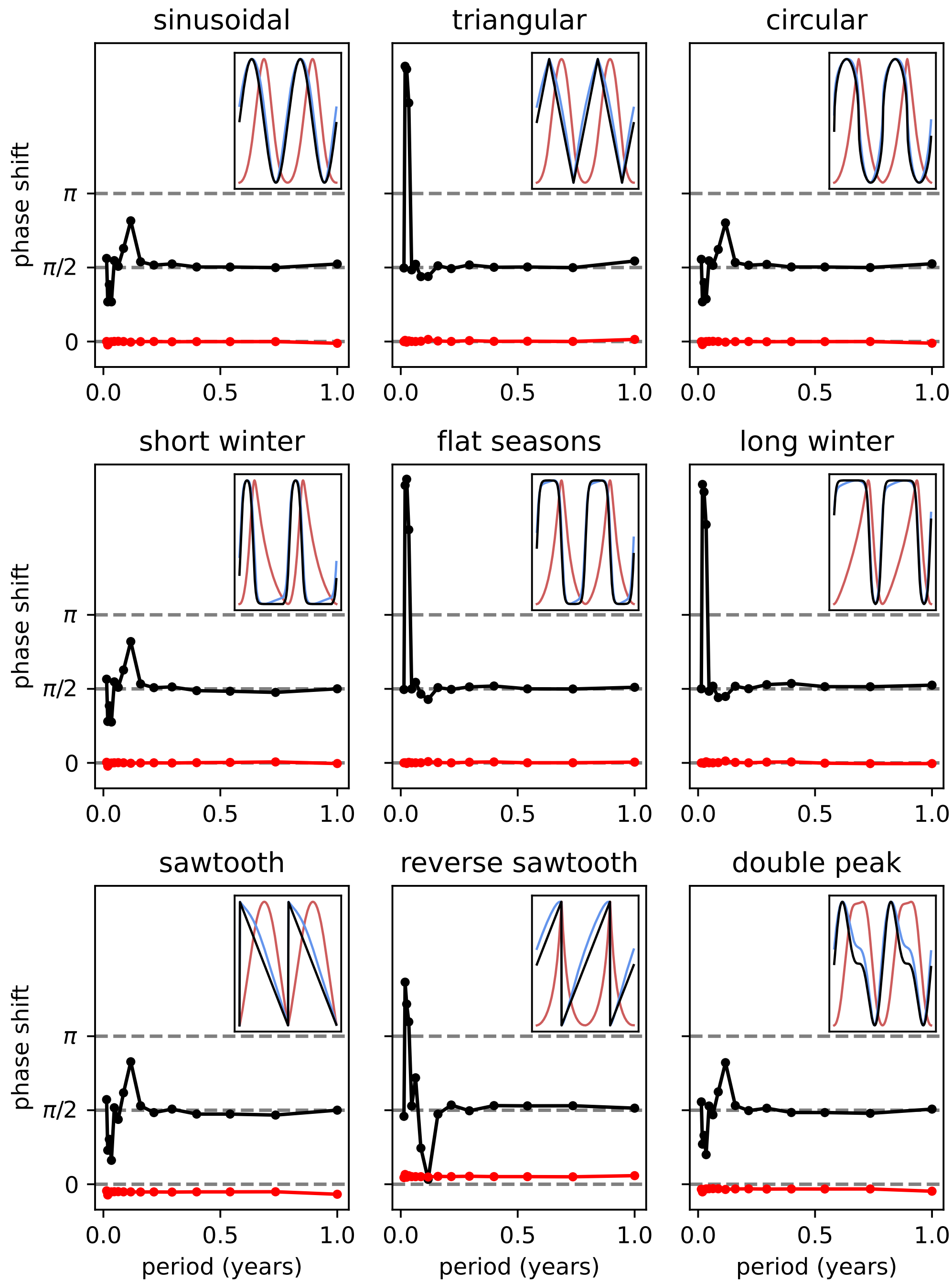

Fig. S5. The phase shift between mitigation and prevalence is robustly $\pi/2$, while mitigation remains in phase with the basic reproduction number, across a wide range of seasonality shapes and periods. In Fig. 2 in the main text, we observed that both the mitigation and the prevalence have one annual wave, with the mitigation peak in winter and the prevalence peak in spring. This corresponds to a phase shift of one season, or $\pi/2$, between the two peaks. To determine whether this phase shift depends on the period or the shape of the seasonal fluctuations, we repeated the analysis for nine different seasonality shapes (sinusoidal, triangular, circular, short winter, flat seasons, long winter, sawtooth, reverse sawtooth, and double peak), each with periodicities ranging from 5 days to 1 year. For all tested shapes and sufficiently long periods, the phase shift between mitigation and prevalence (black line) is close to $\pi/2$, while the phase shift between mitigation and the basic reproduction number (red line) is close to zero, meaning that the bulk timing of the optimal mitigation tracks the seasonality even when their full waveforms differ. To obtain these results, we measured the phase of each signal by projecting onto the Fourier mode at the known periodicity: for a signal $x(t)$ with period T, the phase is given by $\phi = \arg\left(\sum_t x(t)\, e^{-2\pi i t/T}\right)$, and the phase shift between two signals is the difference of their respective phases. The deviations from the asymptotic values for small periodicities are due to numerical errors induced by our time discretization of 1 day. The black line in each inset shows the shape of the seasonality, the red line the typical infection wave, and the blue line the typical mitigation wave, each averaged across different periodicities; visible differences between the black and blue curves illustrate that the mitigation waveform is not a simple rescaling of the seasonality, even though the two are phase-locked at the fundamental.

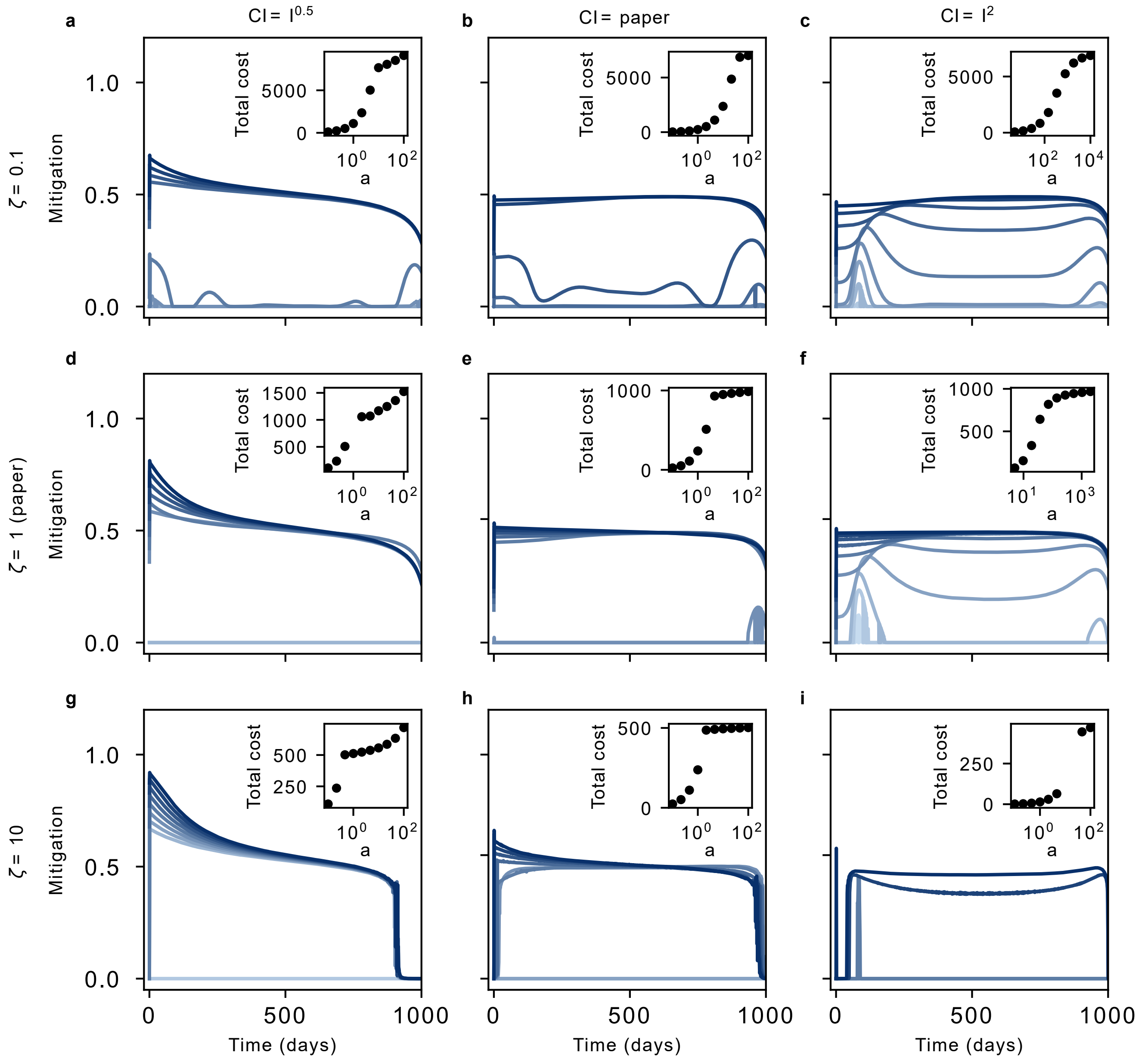

Fig. S6. Optimal mitigation strategies under different infection cost functions and mitigation cost steepness Each panel shows the time series of optimal mitigation $M(t)$ over 1000 days for a range of infection cost scaling values a (color intensity, light to dark), under a SIRS model ($\beta = 0.2$, $\gamma = 0.1$, $\nu = 0.01$, $I_0 = 10^{-4}$). Rows correspond to different values of the mitigation cost steepness parameter ζ ($\zeta = 0.1,\ 1,\ 10$), with $C_M(m) = m/(1 - m^{\zeta})$. Columns correspond to different infection cost functions: $C_I = I^{0.5}$ (sublinear), the one used in the manuscript (linear above hospitalization capacity), and $C_I = I^2$ (quadratic). Insets show total cost as a function of the disease severity a on a log scale. Across most parameter combinations, the optimal solution converges to one of two qualitative regimes: no mitigation ($M \simeq 0$), and maximal mitigation. When $C_I = I^2$, and low value of ζ the mitigation changes smoothly with a. Two data points in the $C_I = I^2, \zeta = 10$ panel, and one in the $C_I = I^{0.5}$, and $\zeta = 1$ are excluded due to optimizer convergence failure.

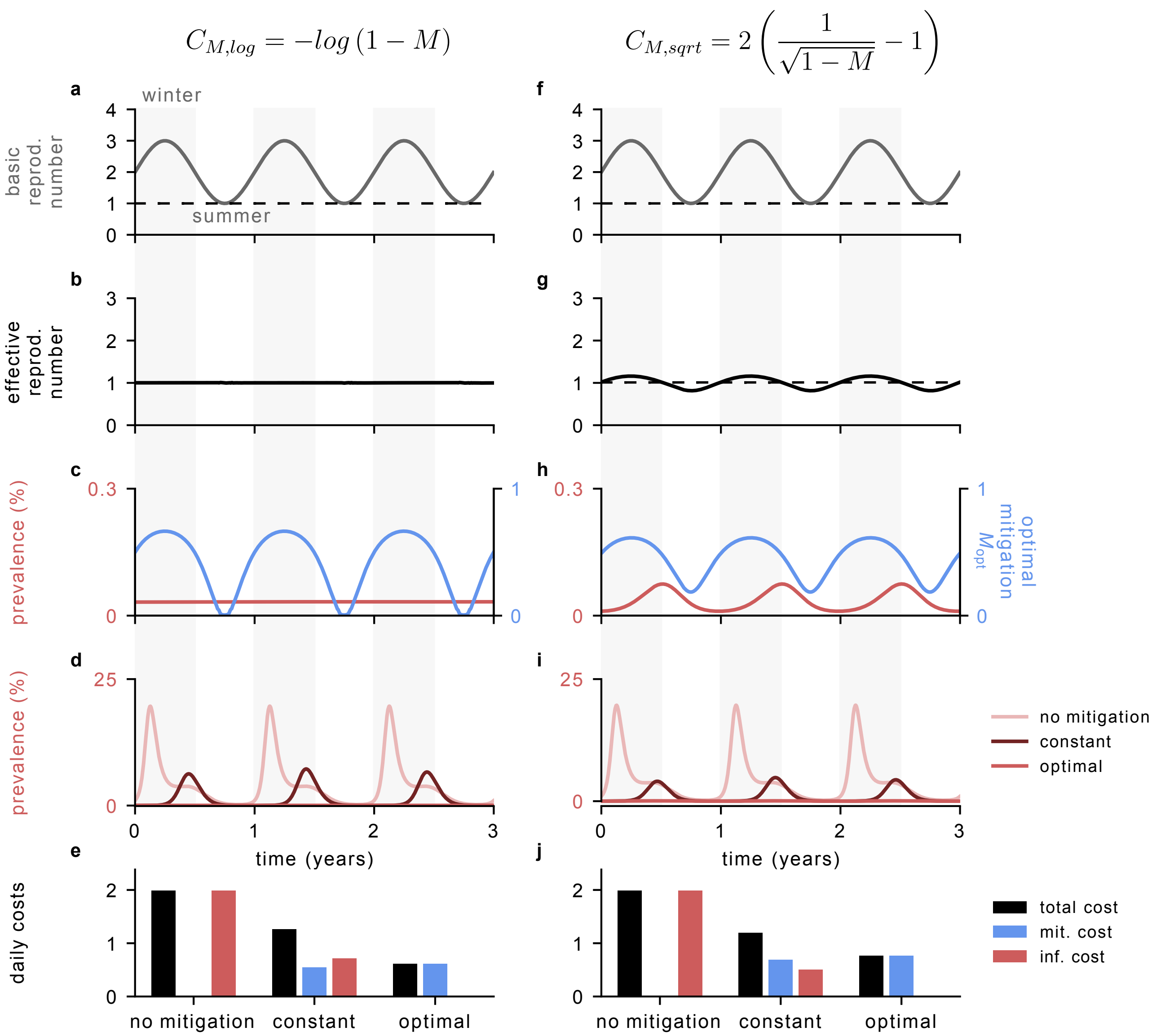

Fig. S7. Comparison between different cost functions in the presence of seasonality. We replicate the scenario from Fig. 2 in the main text using two alternative mitigation cost functions: logarithmic (Eq. (13), panels **a-e**) and square root (Eq. (14), panels **f-j**). With the logarithmic cost function, which grows more slowly than our default (see Fig. S1), mitigation can afford to be strong enough to completely counteract seasonal fluctuations, maintaining $R_{\text{eff}} \approx 1$ throughout the year. This results in approximately constant, low prevalence without seasonal waves (panels **a-e**). The square root cost function produces intermediate results: the effective reproduction number oscillates around 1, creating annual infection waves peaking in spring, similar to our default cost function but with slightly different dynamics (panels **f-j**). These results demonstrate that while the qualitative strategy (counteracting seasonality with mitigation) remains consistent, the achievable level of control depends on how steeply mitigation costs rise.

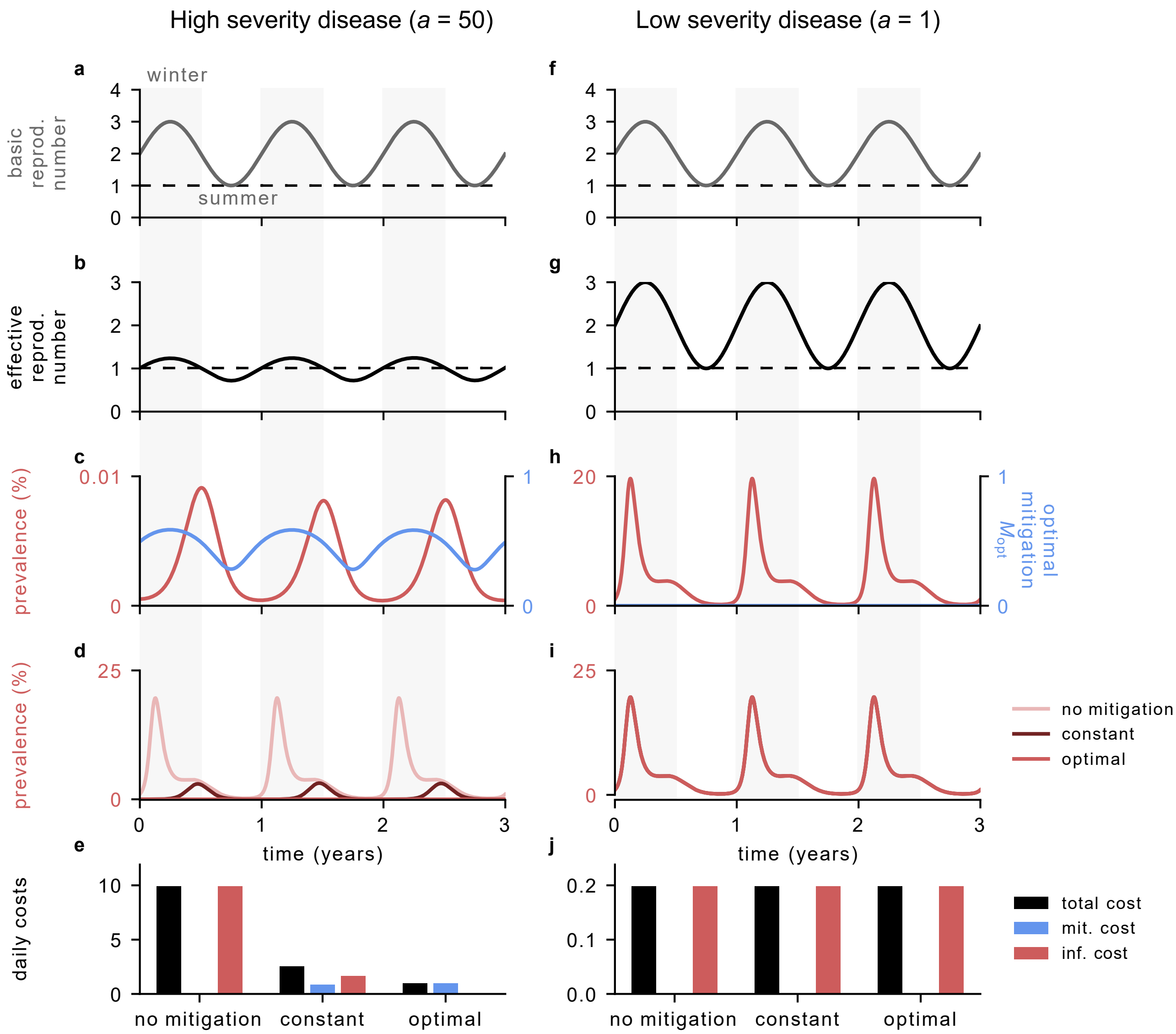

Fig. S8. Comparison between different disease severities a in the presence of seasonality. We extend Fig. 2 in the main text ($a = 10$) to examine how disease severity affects optimal mitigation strategies: $a = 50$ (panels **a-e**) represents a severe disease, and $a = 1$ (panels **f-j**) represents a mild disease. For the severe disease ($a = 50$), the high cost of infections drives strong mitigation that oscillates in phase with the basic reproduction number, keeping the effective reproduction number near 1 and prevalence low. While this results in minimal seasonal variation in prevalence (panel **c**), the infection waves are several orders of magnitude smaller than without mitigation. For the mild disease ($a = 1$), no mitigation is applied and prevalence is high with pronounced seasonal waves. The low infection cost makes tolerating this high prevalence economically optimal. These contrasting outcomes demonstrate how disease severity fundamentally shapes optimal pandemic response: severe diseases require strong, seasonally adapted suppression while mild diseases may tolerate higher infection levels with no control.

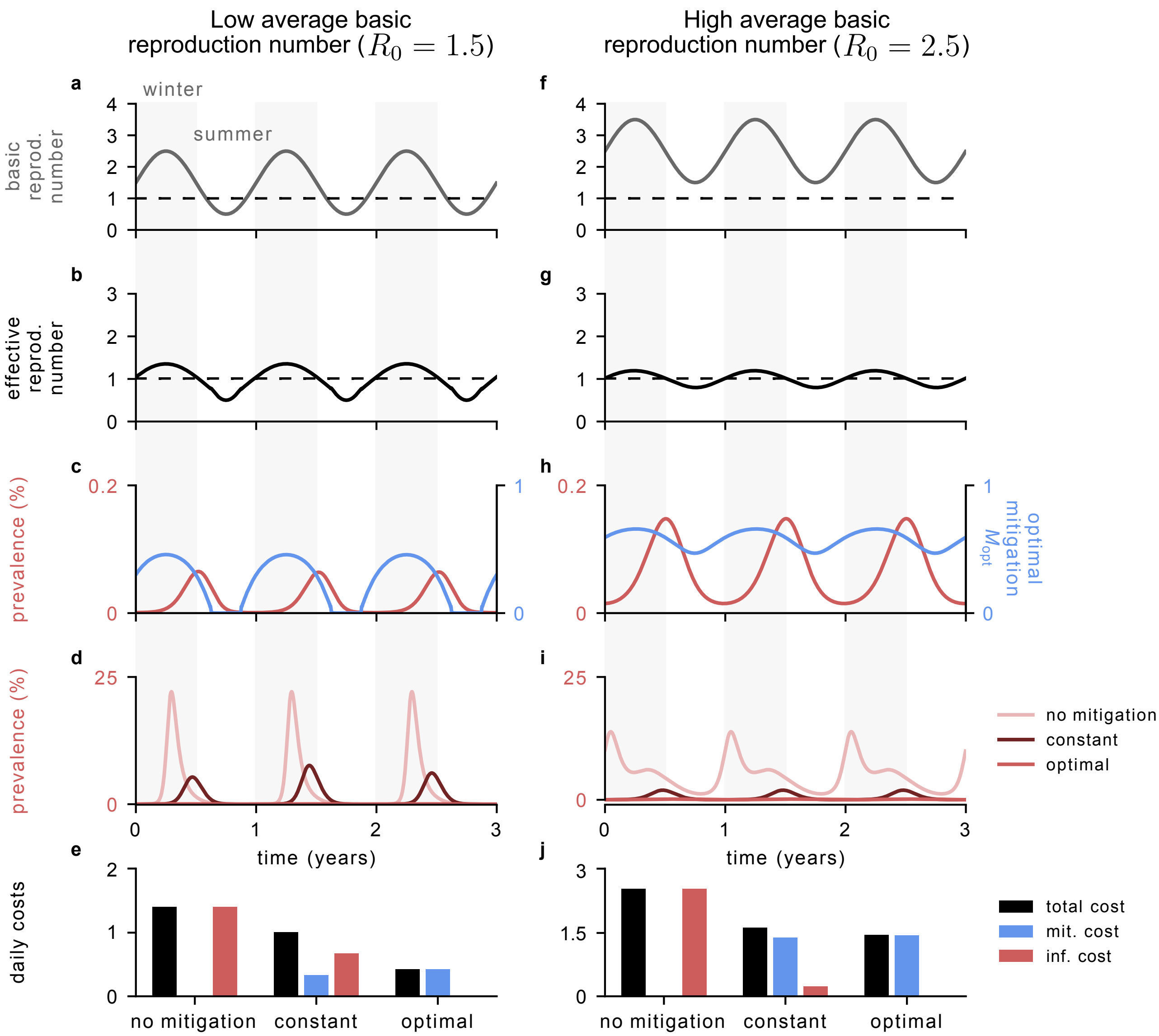

Fig. S9. Comparison between different mean values $R_{0,\text{mean}}$ of the seasonal fluctuations in the basic reproduction number. We examine how the mean basic reproduction number affects optimal mitigation using $R_{0,\text{mean}} = 1.5$ (panels **a-e**) and $R_{0,\text{mean}} = 2.5$ (panels **f-j**), keeping the seasonal amplitude fixed at 1. For reference, Fig. 2 in the main text shows results for $R_{0,\text{mean}} = 2$. For $R_{0,\text{mean}} = 1.5$, the basic reproduction number drops below 1 for approximately one quarter of the year (summer), allowing mitigation to cease entirely during this period. Mitigation peaks in winter when transmission is highest, resulting in a single spring infection wave under optimal control. For $R_{0,\text{mean}} = 2.5$, year-round mitigation is required since even during summer, the basic reproduction number is greater than one. The optimal strategy maintains small oscillations in R_{eff} around 1, producing spring infection waves similar to the default scenario but with higher total costs due to the increased mitigation burden. These results show that the baseline transmission level determines whether seasonal "mitigation holidays" are possible (low $R_{0,\text{mean}}$) or whether continuous control is necessary (high $R_{0,\text{mean}}$).

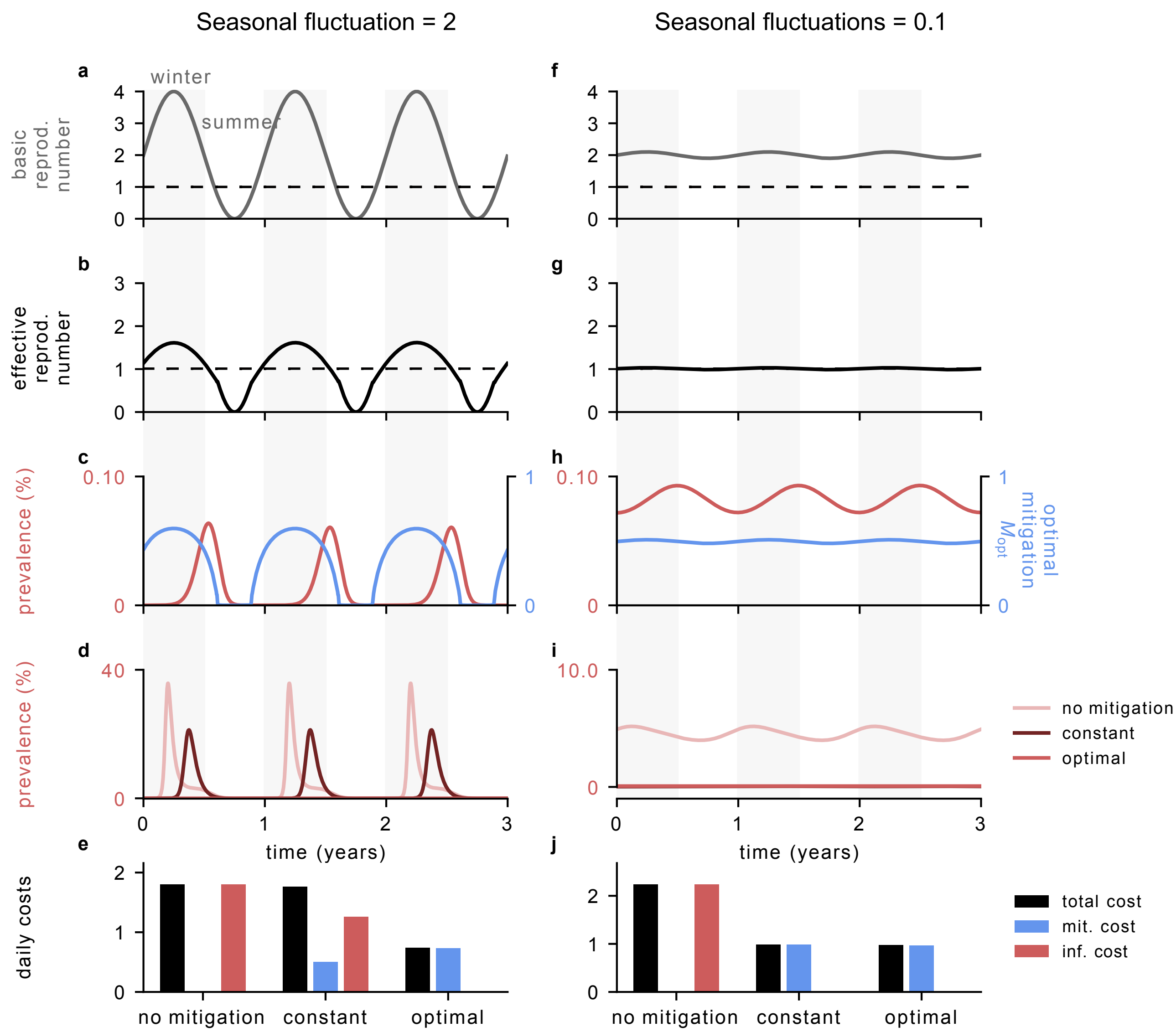

Fig. S10. Comparison between different amplitudes ΔR of the seasonal fluctuations in the basic reproduction number. In Fig. 2 in the main text, we had a mean basic reproduction number of 2 with an amplitude of 1. Here, we will keep the mean basic reproduction number of the seasonal fluctuations at 2 while varying the amplitude. We use $\Delta R = 2$ and $\Delta R = 0.1$ (panels **a-e**, and **f-j**, respectively).

For an amplitude of 2 (panels **a-e**), the basic reproduction number falls below 1 for about a quarter of the year, similar to the case above where we varied the mean basic reproduction number. For the optimal mitigation we get similar results: when the basic reproduction number is below one, the mitigation is zero. Otherwise, it increases, with a peak in winter. For an amplitude of 0.1 (panels **f-j**), the mitigation is nearly constant, as the oscillations of the basic reproduction number are small compared to its mean value (5 %). Consequently, the prevalence exhibits only small oscillations. In conclusion, the amplitude of the seasonality significantly influences the shape of the optimal mitigation, which ranges from nearly constant (low amplitude) to sinusoidal (medium amplitude) to partly zero throughout the year (high amplitude).

$$C_{M,log} = -log\,(1 - M)$$

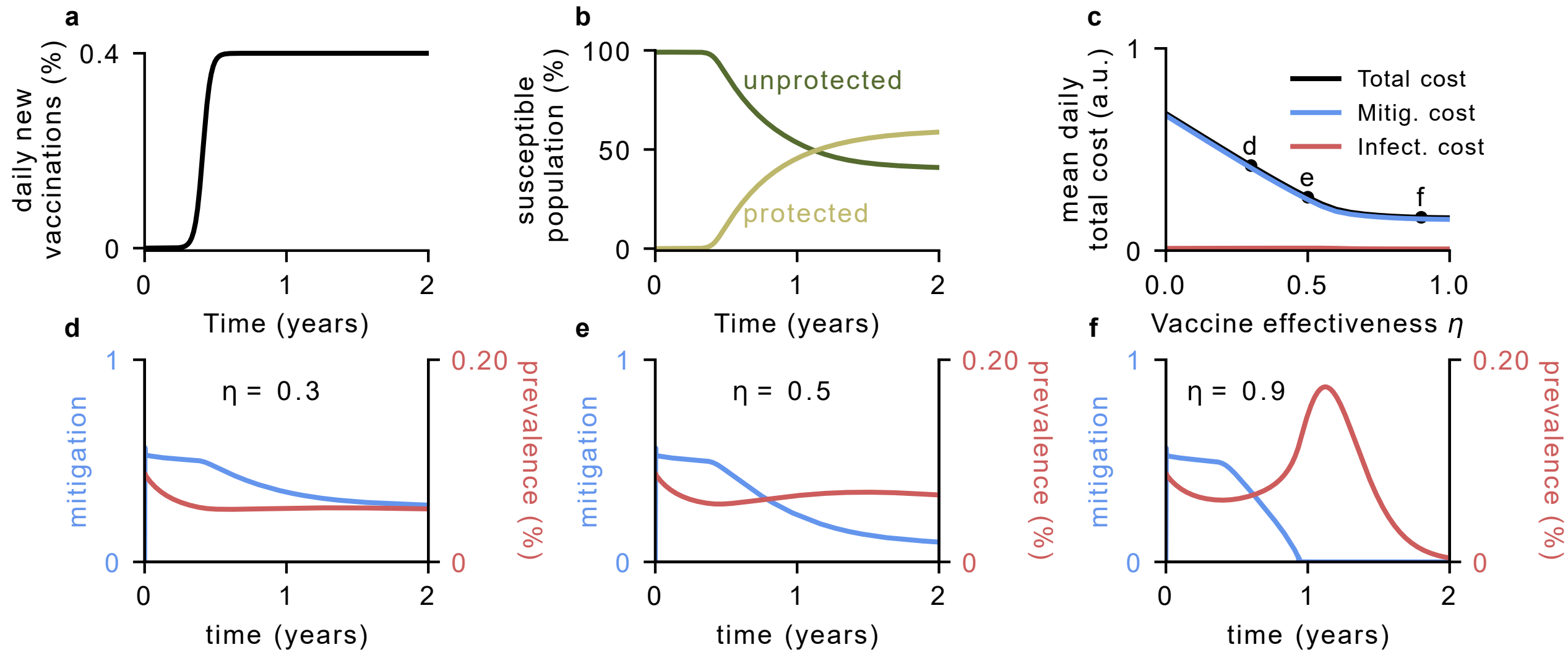

$$C_{M,sqrt} = 2\left(\frac{1}{\sqrt{1-M}} - 1\right)$$

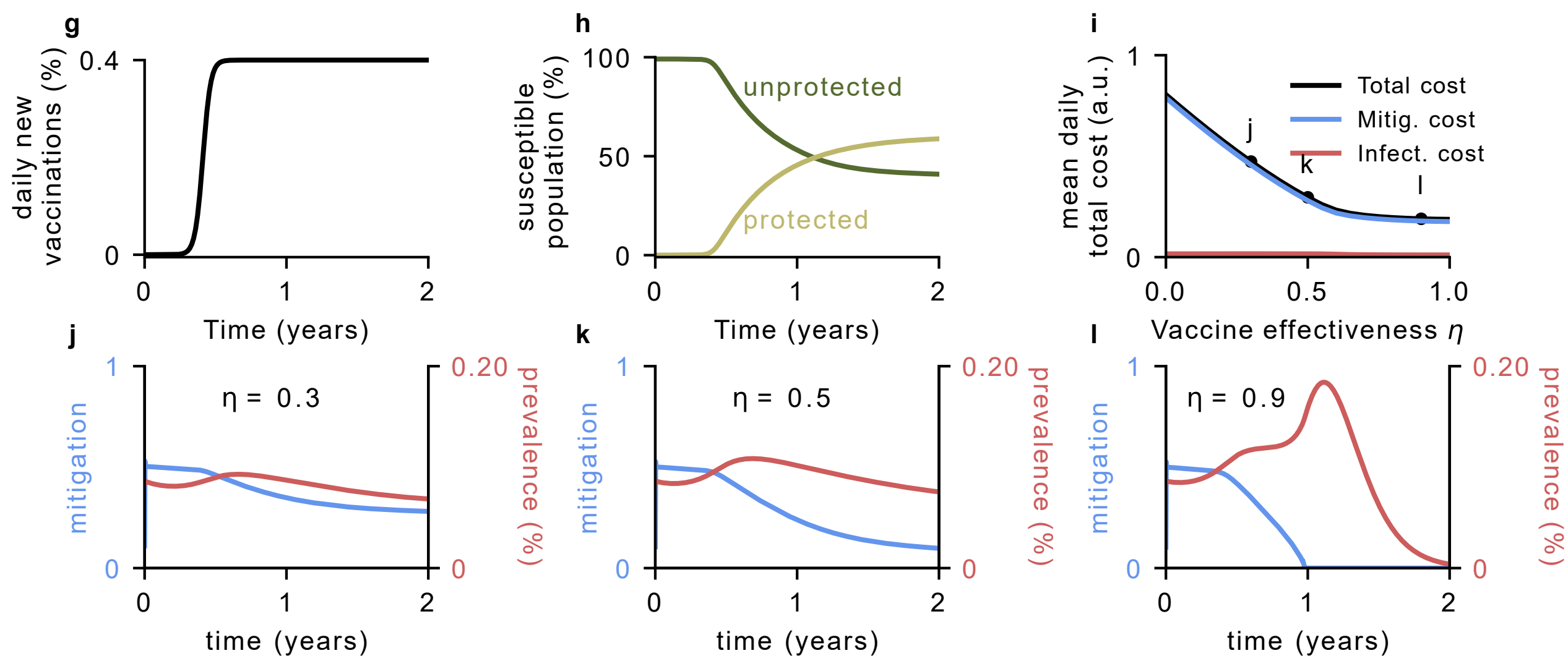

Fig. S11. Comparison between different cost functions in the vaccination scenario. We test the robustness of our vaccination results (Fig. 3 in the main text) using alternative mitigation cost functions: Eq. (13) (panels **a-f**) and Eq. (14) (panels **g-l**), with all other parameters unchanged. The qualitative behavior remains consistent across all cost functions: mitigation decreases during the vaccination campaign, with greater reductions for more effective vaccines. However, the precise dynamics differ between cost functions, leading to variations in prevalence patterns and the timing of transient infection waves.

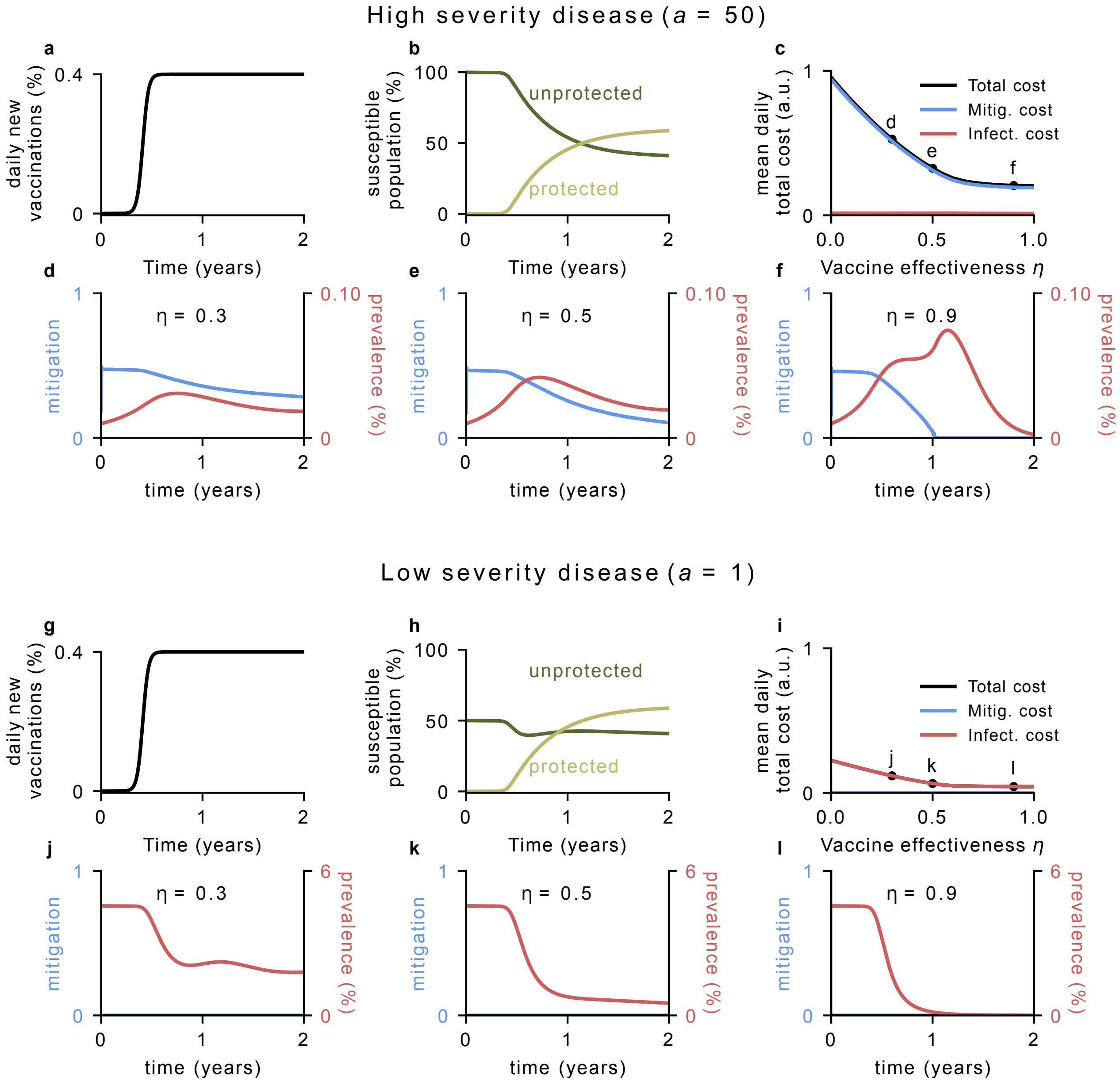

Fig. S12. Comparison between different disease severities a in the vaccination scenario. In Fig. 3 in the main text, we simulated a vaccination campaign with disease severity $a = 10$. Here, we show how the same vaccination scenario for two different severities, $a = 50$ and $a = 1$ (panels **a-f** and **g-l**, respectively), while keeping the other parameters fixed as in Fig. 3 in the main text. For $a = 50$ (panels **a-f**), the high infection cost requires strong mitigation throughout the vaccination campaign, keeping prevalence minimal. Therefore, the total cost is dominated by mitigation rather than infection costs. For $a = 1$, (panels **g-l**), the low infection cost makes mitigation uneconomical, so it remains at zero throughout. Here, total costs are dominated by infection costs, in contrast to the higher severity scenarios. These results align with the results for different disease severities in the other scenarios: for higher severity we get higher mitigation values and lower prevalence, and for less severe diseases we observe that no vaccination is optimal.

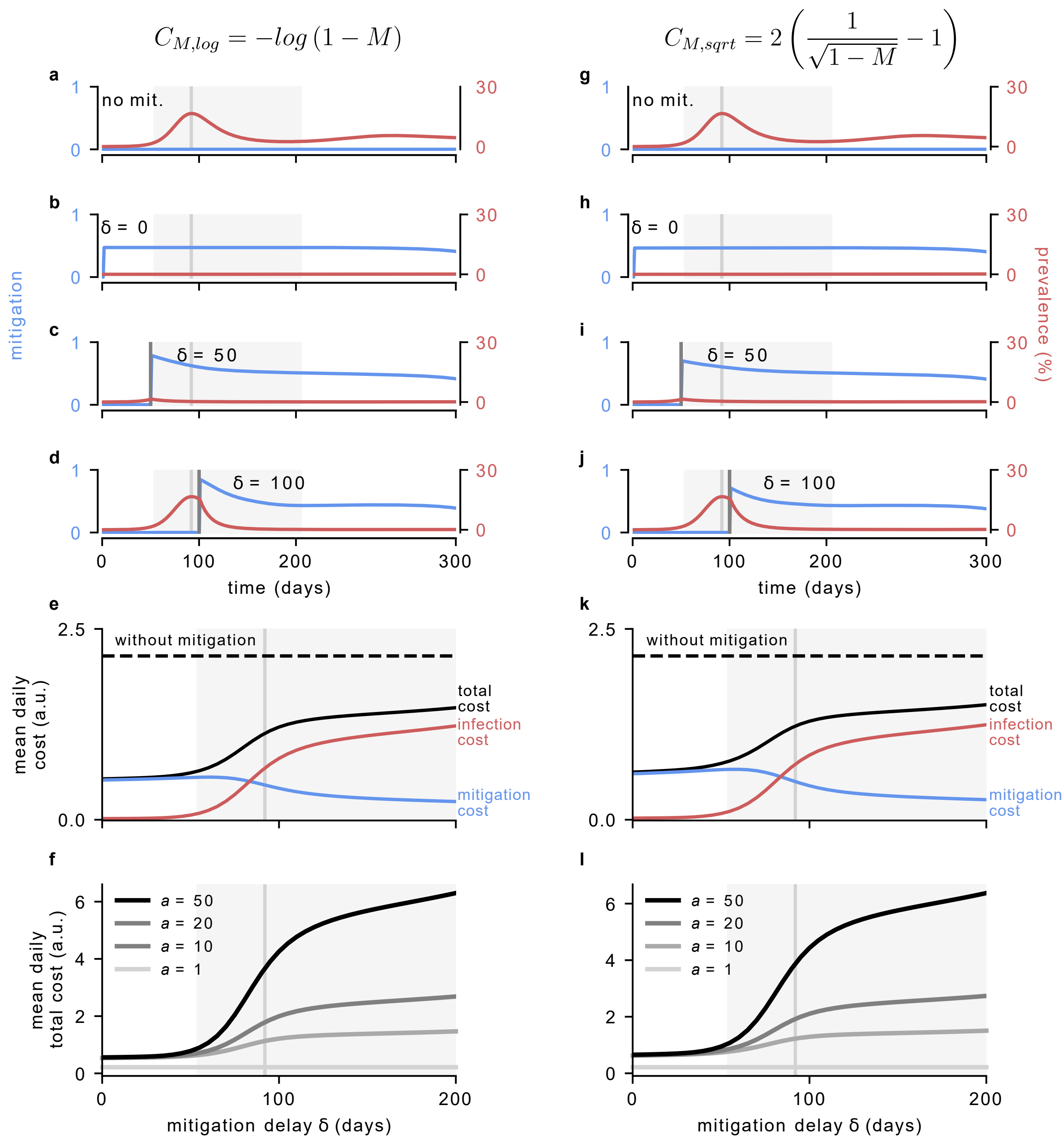

Fig. S13. Comparison between different cost functions in the scenario with delayed mitigation measures. We test how different mitigation cost functions affect the cost of delay using Eq. (13) (panels **a-f**) and Eq. (14) (panels **g-l**), compared to our default in Fig. 4 in the main text. All cost functions produce qualitatively similar results: mitigation begins at high intensity to control the outbreak, then relaxes. Total costs increase monotonically with delay due to accumulating infections during the unmitigated period. While the exact dynamics vary slightly between cost functions, the overall pattern remains robust, demonstrating that our main findings about the cost of delay hold regardless of the specific function chosen for mitigation costs.

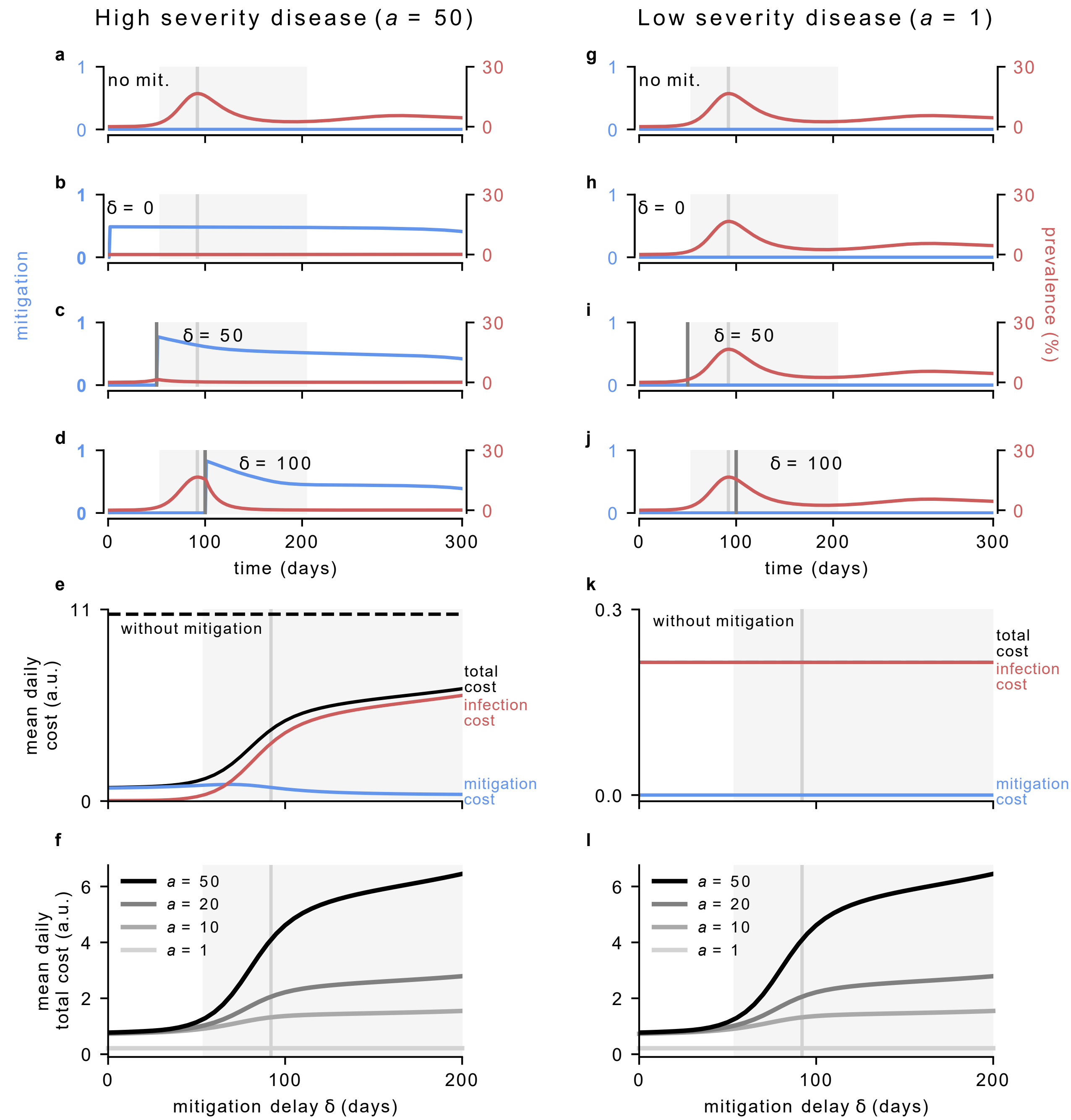

Fig. S14. Comparison between different disease severities a in the scenario with delayed mitigation measures. We examine how disease severity affects the cost of delayed intervention by comparing $a = 50$ (panels **a-f**) and $a = 1$ (panels **g-l**) with our default $a = 10$ shown in Fig. 4 in the main text. For severe disease ($a = 50$, panels **a-f**), delayed mitigation begins at high intensity to rapidly suppress the ongoing outbreak, then relaxes to maintain low equilibrium prevalence. Total costs increase substantially with delay due to the accumulation of infections during the unmitigated period. For mild disease ($a = 1$, panels **g-l**), optimal mitigation remains at zero regardless of delay, as the low infection cost makes intervention uneconomical. Consequently, total costs remain constant with delay and are dominated entirely by infection costs. These results demonstrate that the cost of delay is most pronounced for severe diseases, while mild diseases show no penalty for delayed response since intervention is never optimal.

References

1. X He, et al., Temporal dynamics in viral shedding and transmissibility of COVID-19. *Nat. Medicine* **26**, 672–675 (2020).
2. Y Goldberg, et al., Protection and Waning of Natural and Hybrid Immunity to SARS-CoV-2. *New Engl. J. Medicine* **386**, 2201–2212 (2022) Publisher: Massachusetts Medical Society _eprint: https://www.nejm.org/doi/pdf/10.1056/NEJMoa2118946.
